# Prostate Cancer Patient‐Derived Organoids (PDOs) as Functional Avatars for Antisense Oligonucleotide (ASO)‐Based Personalized Therapy

**DOI:** 10.1002/adhm.202505251

**Published:** 2026-10-11

**Authors:** Thi Thom Tran, Thi Tu Uyen Phan, Michael Baboudjian, Quang Hieu Duong, Ines Sousa, Thi Thuy Le, Virginie Baylot, Sophie Giusiano, Laurent Daniel, Pauline Brige, Benjamin Guillet, Cyrille Bastide, Carmen Garrido, David Taïeb, Thi Khanh Le, Palma Rocchi

**Affiliations:** ^1^ INSERM ERL U1326 CINaM CERIMED CNRS UMR 7325 Aix‐Marseille University Marseille France; ^2^ Department of Urology Hospital Nord, AP‐HM Aix‐Marseille University Marseille France; ^3^ Laboratoire d'Anatomie et Cytologie Pathologiques, Hôpital de la Timone, AP‐HM Marseille France; ^4^ European Center for Research in Medical Imaging (CERIMED) Aix‐Marseille University Marseille France; ^5^ University of Burgundy Europe, INSERM UMR 1231, HSP‐Pathies Centre for Translational and Molecular Medicine Dijon France; ^6^ University of Burgundy Europe Centre Georges‐François Leclerc (CGFL) Dijon France; ^7^ Department of Nuclear Medicine Hôpital de la Timone, AP‐HM, Aix‐Marseille University Marseille France

**Keywords:** antisense oligonucleotides (ASOs), apatorsen (OGX‐427), heat shock protein 27 (HSP27), mRNA targeting, patient‐derived organoids (PDOs), precision medicine, prostate cancer (PC)

## Abstract

Patient‐derived tumor organoids (PDOs) closely preserve the cellular heterogeneity, histopathology, and molecular features of the original tumors, making them powerful preclinical models. Antisense oligonucleotides (ASOs) are an emerging class of precision medicines that modulate gene expression at the RNA level with high target specificity, yet their application in organoid systems remains underexplored. Here, we adapted previously described protocols to establish a biobank of prostate cancer (PC) PDOs, achieving a high initial growth success rate (>90%). Histological and molecular analyses confirmed the preservation of key tumor features. Using these models, we assessed several ASOs, with particular emphasis on OGX‐427 (Apatorsen), an ASO targeting heat shock protein 27 (HSP27) that has been evaluated in phase I/II clinical trials. While OGX‐427 effectively suppressed HSP27 expression and inhibited cell growth in 2D cultures, its efficacy was markedly limited in 3D organoids, likely due to extracellular matrix and structural barriers. Removing Matrigel and applying single‐cell transfection overcame these barriers, achieving efficient ASO uptake, significant target protein downregulation, and robust, dose‐dependent inhibition of organoid growth. These findings underscore the utility of PDOs for evaluating ASO‐based therapies and their potential to accelerate precision oncology strategies for PC and other malignancies.

## Graphical abstract

Prostate patient‐derived organoids (PDOs) were established from TURP and biopsy specimens with >90%  success and retain key features of their tissues of origin, making them functional avatars for antisense oligonucleotide (ASO) therapy . Transfecting single cells outside the Matrigel matrix and then reconstituting organoids overcomes the 3D delivery barrier, achieving efficient ASO uptake, target knockdown and dose‐dependent growth inhibition. Created in BioRender. L, T.K (2026) http://BioRender.com/6wa8h83

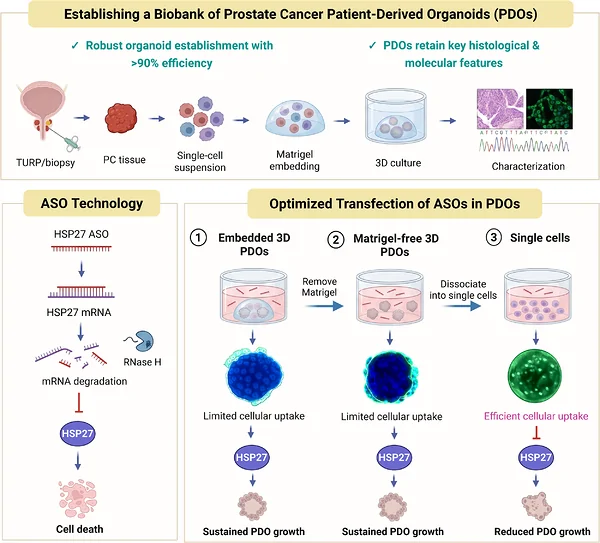

## 1 Introduction

Prostate cancer (PC) is the second most commonly diagnosed cancer in men, with an estimated 1.46 million new cases and 396 792 deaths worldwide in 2022 [1]. Androgen deprivation therapy (ADT) remains the cornerstone of treatment for metastatic PC [2]. In metastatic castration‐sensitive PC (CSPC), ADT is often administered in combination with androgen receptor (AR) pathway inhibitors and/or taxane‐based chemotherapy. Recently reported phase III trials have demonstrated a survival benefit of upfront triplet therapy with ADT, docetaxel plus either abiraterone acetate or darolutamide when compared to ADT plus docetaxel alone [3]. Despite initial responses to treatment, most patients with advanced or metastatic PC eventually develop castration‐resistant prostate cancer (CRPC), for which the efficacy of available therapeutics (e.g., taxanes, ^177^Lu‐PSMA radioligand therapy, and PARP inhibitors in *BRCA1/2 or ATM* ‐altered tumors) remains limited. In parallel with the development of new therapies and their evaluation in clinical trials, significant research efforts have been directed toward understanding the molecular mechanisms responsible for therapy resistance and identifying effective strategies to counteract them [4].

In recent years, antisense oligonucleotide (ASO) technology has emerged as an innovative therapeutic approach in precision medicine. The field has gained momentum with US Food and Drug Administration (FDA) and European Medicines Agency (EMA) approvals of 13 ASO‐based drugs for various disorders, including cardiovascular, metabolic, neurological, and infectious diseases [5]. ASOs are short, synthetic chemically modified single‐stranded oligonucleotides that bind to complementary RNA sequences, allowing for the modulation of targeted mRNA transcripts with high specificity [6, 7, 8]. ASO‐based investigational drugs offer several benefits compared to small molecules and antibodies, such as flexibility in design due to Watson–Crick base recognition, optimized synthesis protocols, and comprehensive quality control during development [9]. A notable example is the rapid translation (less than one year) of milasen from proof‐of‐concept to clinical use, showcasing ASO‐based precision medicine's potential to improve patients' quality of life by reducing disease severity [5]. ASOs can therefore be employed both as patient‐customized medicines and, more broadly, to treat large patient populations.

Efforts to elucidate PC development and treatment response have been significantly impeded by the lack of models that faithfully recapitulate the disease's considerable heterogeneity. Recently, patient‐derived organoids (PDOs) have emerged as a robust and versatile platform for precision medicine, as they retain key cellular, structural, and molecular features of the original tumors [10, 11]. These models enable high‐throughput drug screening and functional testing, thereby facilitating the rapid translation of individualized therapeutic strategies into clinical settings. A growing number of clinical trials worldwide are leveraging PDOs in correlative studies to improve treatment stratification and outcomes [12]. Although numerous clinical trials have involved the use of organoids, the most commonly investigated cancer types in these trials are breast cancer, pancreatic cancer, and colorectal cancer. The widespread clinical application of PDOs in PC has been limited by the technical challenges in their generation and long‐term maintenance [13, 14]; consequently, very few PC biobanks have been created to date [6, 14, 15, 16]. Most long‐term PC PDO lines reported so far have been derived from metastatic biopsies or circulating tumor cells, and the success rate for generating PDOs from advanced PC remains low (approximately 16%–25%), markedly lower than those for bladder, breast, or colorectal cancers, which range from 70% to 90% [13, 17].

Establishing PDOs from primary PC tissues would provide a valuable platform to study early disease progression and to develop personalized treatment strategies prior to metastasis. Although ASOs hold significant therapeutic promise, their application in clinically relevant three‐dimensional (3D) PDO models remains limited. In this study, we established a biobank of organoids derived from primary PC tissues, most of which were obtained through transurethral resection of the prostate (TURP). Using previously described organoid culture protocols, with an overnight collagenase digestion applied to all specimens, we achieved a high initial growth success rate (>90%). The generated PDOs retained the histological architecture and key molecular features of their corresponding tumors. We further identified major barriers to ASO delivery within 3D Matrigel cultures and optimized a protocol for evaluating ASO therapeutics in PDOs. Our findings highlight the feasibility of using primary PC PDOs for ASO testing and support their utility as a platform for precision medicine in PC and other malignancies.

## 2 Results

### 2.1 Establishment of Patient‐Derived Organoids (PDOs) From Prostate Cancer Tissues

Our primary goal was to establish a biobank of patient‐derived organoids (PDOs) from prostate tissue specimens. Between May 2023 and April 2025, 27 prostate tissue specimens were collected under AP‐HM Biological Resources Center authorization (#AC‐2023‐5572 and #AC‐2024‐6402). Specimens were predominantly obtained via TURP, with recent additions from needle biopsies (Figure 1A, Table S1). Histopathological analysis confirmed that >70% of the cells in tumor samples were malignant. The biobank currently includes samples from 12 patients with benign prostatic hyperplasia (BPH), 14 with castration‐sensitive PC (CSPC), and 1 with castration‐resistant PC (CRPC) (Figure 1B). Of the 27 samples, 21 were processed for organoid culture using previously established protocols [13, 18]. An overnight collagenase type II digestion (5 mg/mL), recommended for human prostate tissue [18], was applied to all specimens, including tumor‐containing TURP specimens and needle biopsies, whereas a short (1 h) digestion is usually used for PC biopsies in previous studies [13, 18]. Using this approach, organoids were successfully generated from 19 of the 21 samples (90.5%; 16/17 TURP and 3/4 biopsy samples), achieving a high success rate for initial organoid growth (Figure 1C).

**FIGURE 1:**
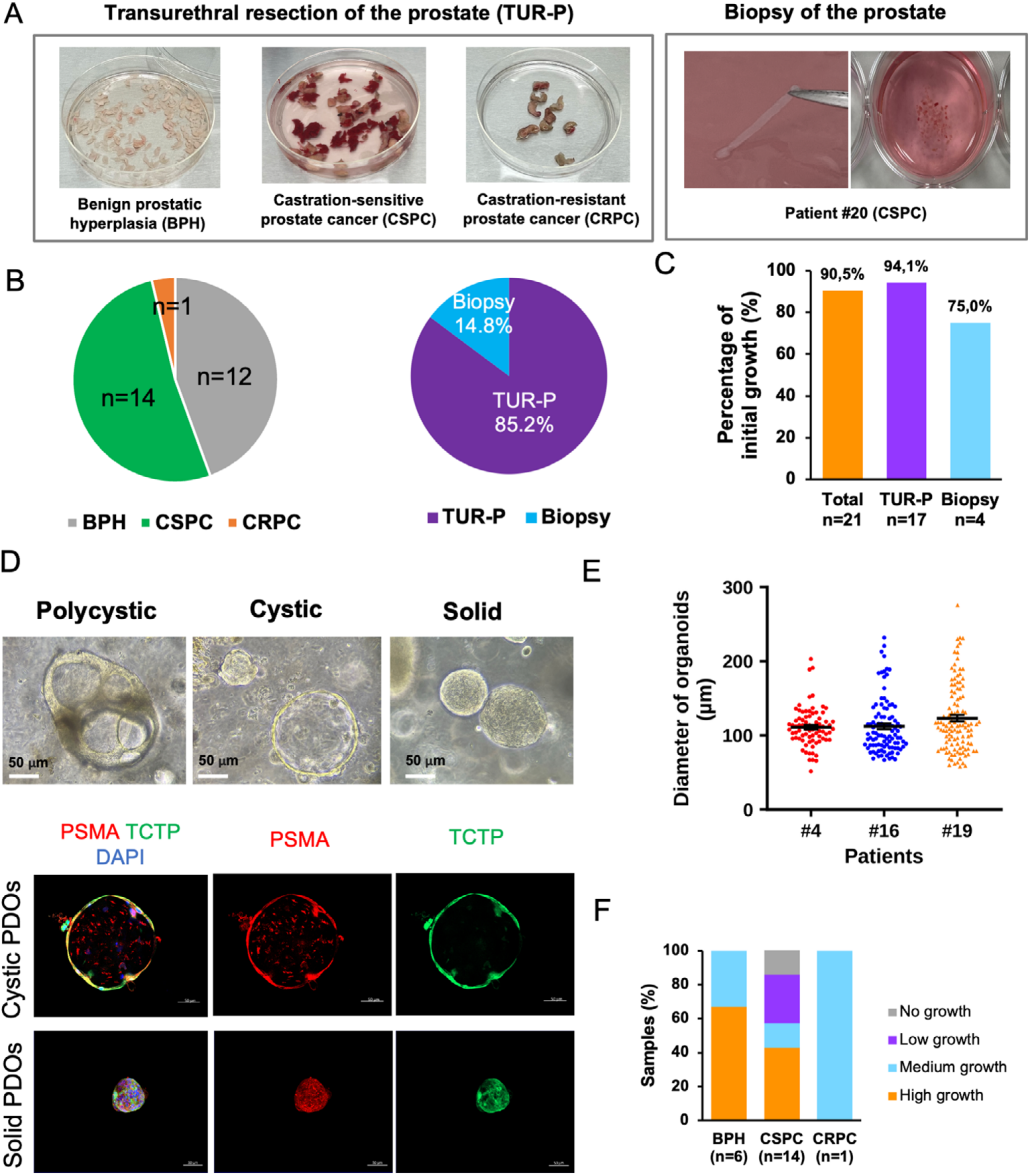
Establishment of a biobank of patient‐derived organoids (PDOs) from prostate tissue specimens. (A) Representative macroscopic images of fresh prostate tissue specimens before processing for organoid generation, obtained by transurethral resection of the prostate (TURP) from patients with benign prostatic hyperplasia (BPH), castration‐sensitive prostate cancer (CSPC), and castration‐resistant prostate cancer (CRPC), and by prostate biopsy (Patient #20, CSPC). Histopathological analysis confirmed that more than 70% of the cells in tumor samples were malignant. (B) Distribution of collected specimens (n=27) by pathology ( BPH, *n* = 12; CSPC, *n* = 14; CRPC, *n* = 1), and by tissue source (TURP, *n* = 23; biopsy, *n* = 4). (C) Percentage of samples showing initial organoid growth: overall, 90.5% (19/21; BPH, *n* = 6; CSPC, *n* = 14; CRPC, *n* = 1); TURP, 94.1% (16/17); biopsy, 75.0% (3/4). (D) Representative brightfield images of three main organoid morphologies (polycystic, cystic, and solid), which co‐existed within the same culture; solid organoids were predominant. Scale bars, 50 µm. Immunofluorescence images show the expression of prostate‐specific membrane antigen (PSMA, red) and translationally controlled tumor protein (TCTP, green); nuclei were counterstained with DAPI (blue) in organoids from Patient #4. (E) Diameter of organoids derived from Patient #4 (BPH; *n* = 77 organoids) and Patients #16 (*n* = 100) and #19 (*n* = 115) (CSPC), measured at passage 0 and/or 1.  Each dot represents one organoid; bars show mean ± SEM. Diameters ranged from approximately 50 to 275 µm. (F) Proportion of samples according to organoid formation efficiency (OFE) at passage 0, by pathology: high (>100 organoids/dome), medium (50–100), low (<50), or no growth. BPH samples showed higher efficiency than tumor‐derived samples (Table S2). Organoids from tumor tissues were maintained for 2–3 passages (up to 5 for Patient #16), whereas BPH‐derived organoids were maintained for up to 5 passages.

Three distinct organoid morphologies were observed: polycystic, cystic, and solid (Figure 1D). Organoid diameters ranged from 50 to over 200 µm within individual cultures (Figure 1E). Generation efficiency varied across patients, with higher yields consistently observed in BPH‐derived tissues compared to tumor tissues (Figure 1F, Table S2). Biopsy‐derived samples produced fewer organoids (<50 organoids per dome), largely due to limited starting materials.

While most organoids from tumor tissues could be maintained for 2–3 passages (up to 5 for Patient #16) (7–21 days per passage), their proliferation rate decreased markedly after the second passage. In contrast, BPH‐derived organoids showed higher initial yields, more robust passaging efficiency, and were sustained in culture for up to 5 passages. Notably, organoids could also be successfully generated from frozen cells, though optimal viability was observed when using freshly isolated or P0 cells (Table S2).

### 2.2 PDOs Faithfully Recapitulate the Histological and Molecular Characteristics of Their Corresponding Tissue of Origin

To evaluate how accurately PDOs reflect their original prostate tissues, we studied the morphological and molecular features of matched tissues and PDOs from four patients (#08, BPH; #19, #23 and #24, CSPC). Hematoxylin‐Eosin‐Saffron (HES) staining showed that the organoids’ overall structure and histopathological characteristics closely mirrored those of the original tissues (Figure 2A).

**FIGURE 2:**
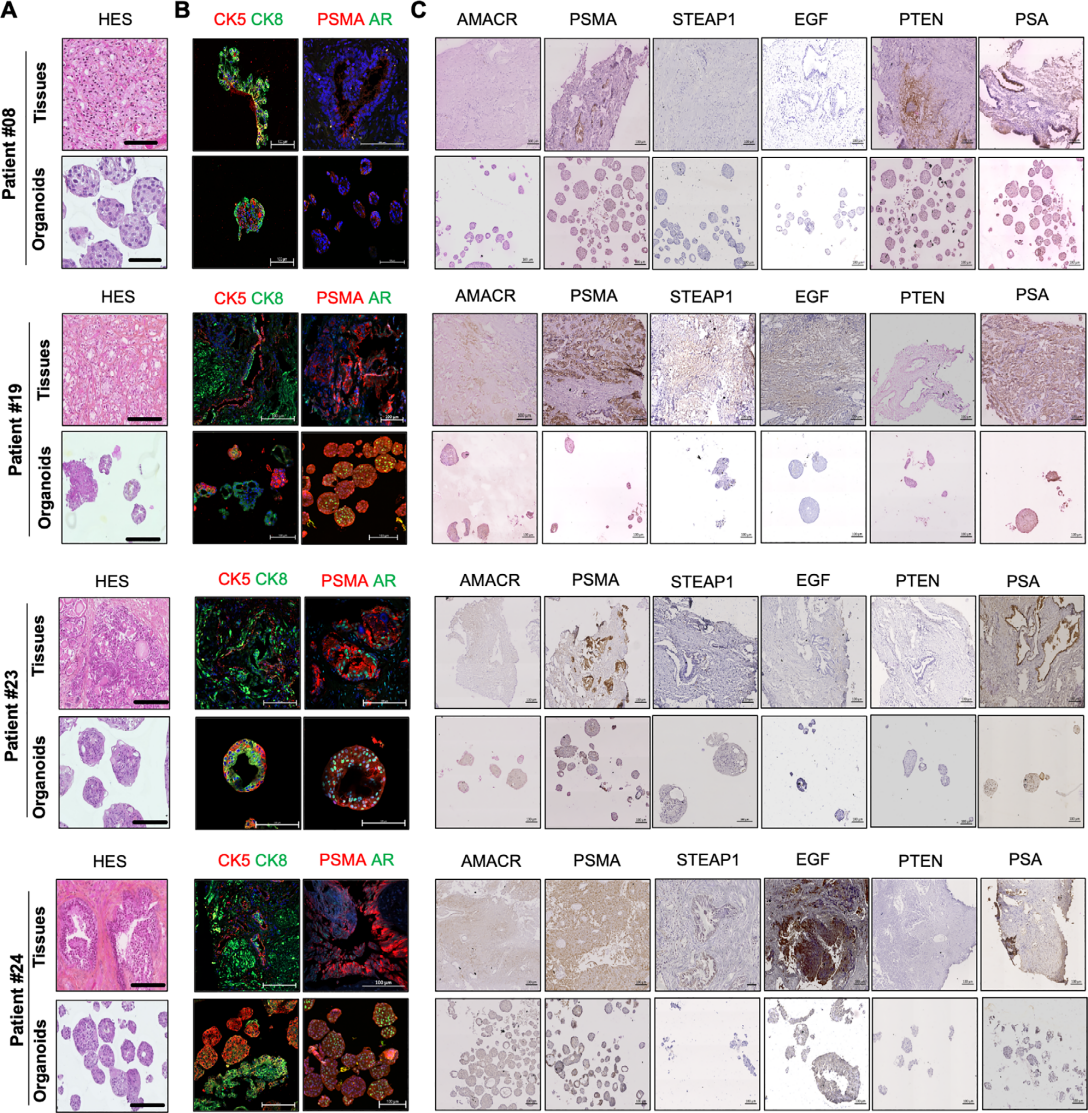
Patient‐derived organoids (PDOs) recapitulate the histological and molecular features of their tissues of origin. Matched tissues (upper rows) and PDOs (lower rows) from four patients. (A) Hematoxylin‐eosin‐saffron (HES) staining shows that the overall architecture of the PDOs resembles that of their tissues of origin. (B) Immunofluorescence staining for cytokeratin 5 (CK5, red) and cytokeratin 8 (CK8, green) reveals the preservation of prostate epithelial identity, with distinct populations of CK5‐positive basal cells and CK8‐positive luminal cells, reflecting the cellular heterogeneity of the native prostate epithelium. CK8‐positive luminal cells are often organized around glandular‐like lumens, surrounded by CK5‐positive basal cells, mirroring the zonal structure of the original tissue. Immunofluorescence staining for PSMA (red) and AR (green) is also shown; nuclei were counterstained with DAPI (blue). (C) Immunohistochemistry for key PC markers, including prostate‐specific membrane antigen (PSMA), prostate‐specific antigen (PSA), alpha‐methylacyl‐CoA racemase (AMACR), epidermal growth factor (EGF), six transmembrane epithelial antigen of the prostate 1 (STEAP1), and phosphatase and tensin homolog (PTEN) . Patient #08, BPH; Patient #19, CSPC, Gleason score, 9 (5+4); Patient #23, CSPC, Gleason score, 7 (4+3); Patient #24, CSPC, Gleason score, 9 (5+4) (Scale bar = 100 µm).

Immunofluorescence analysis revealed that PDOs express both basal (CK5) and luminal (CK8) epithelial markers, demonstrating preservation of prostate epithelial identity. Some organoids co‐expressed CK5 and CK8, while others contained distinct populations expressing only one marker (Figure 2B), reflecting the heterogeneity typical of native prostate epithelium. In BPH‐derived organoids, CK8‐positive luminal cells were often arranged around glandular‐like lumens and surrounded by CK5‐positive basal cells, mimicking the bilayered architecture of the original tissues. Additionally, PDOs express androgen receptor (AR) [Correction added on 21 October 2026, after first online publication: The sentence was updated to “Additionally, PDOs express androgen receptor (AR)”.], prostate‐specific membrane antigen (PSMA) (Figure 2B), and immunohistochemistry for prostate‐specific antigen (PSA), alpha‐methylacyl‐COA racemase (AMACR), epidermal growth factor (EGF), six transmembrane epithelial antigen of the prostate 1 (STEAP1), and phosphatase and tensin homolog (PTEN) showed expression pattern broadly concordant with those of the matched tissues, although staining intensity varied for some markers (Figure 2C). It should be noted that the histological and partial molecular characterization of PDOs derived from Patients #19 and #24 was previously reported in our earlier study [19, 20]. In the present study, we provide a more comprehensive molecular characterization by including additional clinically relevant markers, namely STEAP1, EGF, PTEN, and PSA.

To further evaluate the preservation of tumor‐associated molecular alterations, Sanger sequencing was performed on genomic DNA extracted from PDOs. Next‐generation sequencing (NGS) analyses conducted on the corresponding patient tumors as part of routine clinical practice by the Medical Biology Laboratory, AP‐HM (Marseille Public University Hospitals), Marseille, France, identified likely pathogenic variants in Patients #3, #13, #21, and #24 (Table S1). Among these cases, organoids were successfully established from the tumors of Patients #3, #21, and #24. However, mutation validation could not be performed in PDOs from Patients #3 and #21 because of insufficient material for downstream analyses, due to failed recovery after cryopreservation and limited organoid yield (Table S2). Consequently, mutation validation was only feasible in the PDOs derived from Patient #24. Sanger sequencing confirmed the heterozygous CHEK2 NM_007194.4:c.319+1G>A variant, detected by NGS in the parental tumor with a variant allele frequency of 46.15%, in the PDO derived from Patient #24 (Figure 3A). These findings indicate that the tumor‐associated genetic alteration was retained in the corresponding PDOs. [Correction added on 21 October 2026, after first online publication: The preceding sentence was updated.]

**FIGURE 3:**
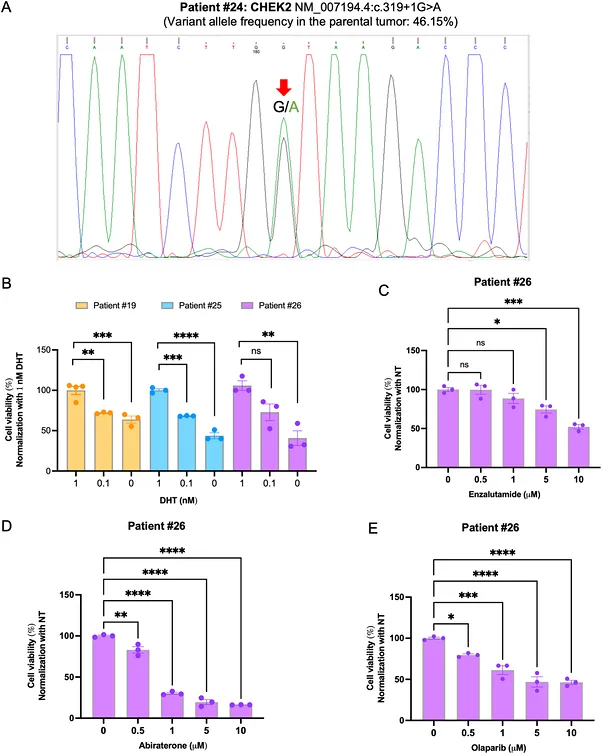
Genetic validation, androgen dependence, and drug responses of prostate cancer (PC) patient‐derived organoids. (A) Sanger sequencing chromatogram showing the detection of the **CHEK2 c.319+1G>A** mutation in the PDO derived from Patient #24. The mutated nucleotide is indicated by an arrow. The mutation was previously identified by clinical NGS analysis of the corresponding parental tumor (variant allele frequency, 46.15%). (B) PDOs derived from three CSPC patients (#19, #25, and #26) show reduced viability under low (0.1 nM) and androgen‐deprived (0 nM) conditions compared to standard 1 nM dihydrotestosterone (DHT), highlighting the androgen dependence of these models. (C–E) Viability of PDOs from Patient #26 treated with the indicated drugs for 2 days in the presence of 1 nM DHT decreased in a dose‐dependent manner; values are normalized to vehicle‐treated controls (NT, 0 µM). Data are expressed as mean ± SEM (*n* = 3 technical replicates per condition; *n* = 4 for Patient #19 at 1 nM DHT). Statistical significance was determined by ordinary one‐way ANOVA for each patient (B) or each drug (C–E), followed by Dunnett's multiple comparisons test versus 1 nM DHT (B) or untreated controls (0 µM; C–E). A *p*‐value < 0.05 was considered statistically significant. *p < 0.05, **p < 0.01, ***p < 0.001, ****p < 0.0001. ns: not significant. Patient #19, CSPC, Gleason score, 9 (5+4); Patient #24, CSPC, Gleason score, 9 (5+4); Patient #25, CSPC, Gleason score, 10 (5+5); Patient #26, CSPC, Gleason score, 10 (5+5).

To assess the functional relevance of androgen signaling in these PDOs, we evaluated the growth of organoids derived from three patients with CSPC (#19, #25, and #26) under varying dihydrotestosterone (DHT) concentrations. As expected, organoid viability was significantly reduced under low (0.1 nM) and androgen‐deprived (0 nM) conditions compared to the standard 1 nM DHT condition, confirming their dependency on androgen signaling (Figure 3B). Of note, androgen dependence was functionally assessed only in PDOs from these three patients and was not systematically evaluated in the remaining PDO samples. In addition, PDOs from Patient #26 showed dose‐dependent sensitivity to the androgen receptor pathway inhibitors enzalutamide and abiraterone, and to the PARP inhibitor olaparib (Figure 3C–E). Collectively, these results show that the established PDOs preserve key histological, molecular, and functional features of the parental prostate tumors. Their ability to maintain prostate epithelial marker expression, selected tumor‐associated genetic alterations, and clinically relevant drug responses supports their utility as a patient‐derived platform for disease modeling and therapeutic testing.

### 2.3 The 3D Architecture of Spheroids and PDOs, Combined With the Presence of Matrigel, Significantly Impairs ASO Delivery

To evaluate ASO efficiency in PDOs, we first selected OGX‐427 (Apatorsen), an ASO targeting heat shock protein 27 (HSP27) mRNA that has been investigated as an anticancer agent in different clinical trials [21, 22]. Our previous studies identified HSP27 as a critical driver of CRPC progression [23, 24]. HSP27 promotes CRPC through multiple mechanisms, including the stabilization of its key oncogenic partners such as eIF4E, TCTP, Menin, and DDX5, by protecting them from proteasomal degradation [25, 26, 27, 28, 29, 30, 31].

Immunohistochemistry and western blot analysis showed that HSP27 was expressed in PDOs, as in their matched tissues, from both BPH and PC patients (Figure 4A,B). In tissues, HSP27 protein levels tended to be higher in PC than in BPH samples; this difference was attenuated in PDOs, as BPH‐derived PDOs showed higher HSP27 levels than their tissues of origin (Figure 4B), possibly reflecting stress‐induced HSP27 expression under culture conditions. In addition, organoids derived from PC tissue exhibited significantly higher HSP27 mRNA expression compared to those derived from BPH (2.5‐fold; *p* < 0.05; Figure 4C).

**FIGURE 4:**
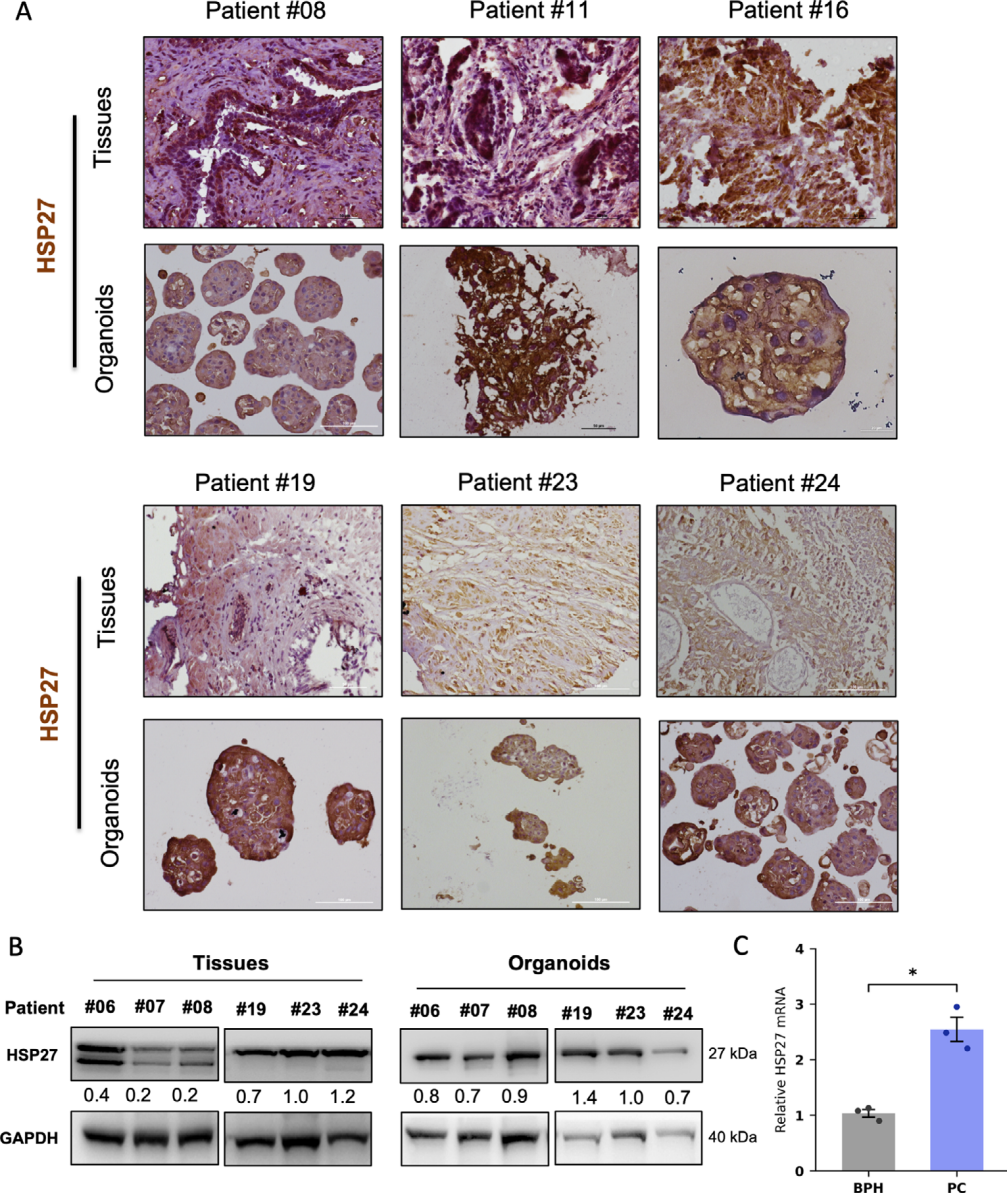
HSP27 is expressed in prostate patient‐derived organoids (PDOs) and their matched tissues. (A) Representative HSP27 immunohistochemistry of matched tissues and PDOs from one BPH patient (#08) and five prostate cancer (PC) patients (#11, #16, #19, #23, and #24). Scale bars as indicated. (B) Western blot analysis of HSP27 in matched tissues and PDOs from three BPH patients (#06, #07, and #08) and three PC patients (#19, #23, and #24); GAPDH, loading control. Numbers below the blots indicate HSP27/GAPDH band intensity ratios quantified with ImageJ. (C) RT‐qPCR showed that HSP27 mRNA levels were higher in prostate cancer‐derived organoids than in those derived from BPH. Each dot represents one patient (mean of the organoid passages analyzed): BPH, *n* = 3 patients (#06, #07, #08); PC, *n* = 3 patients (#05, #09, #11). Patient #04 was excluded from this analysis because, although the resected tissue was benign, the patient had previously received multiple treatments for castration‐resistant PC (Table S1). Data are expressed as mean ± SEM (standard error of the mean). Significant differences between groups were determined using a two‐tailed *t*‐test with Welch's correction (*P* = 0.014). *p < 0.05. [Correction added on 21 October 2026, after first online publication: The sentence was updated to “standard error of the mean”.]

OGX‐427 was previously shown to suppress HSP27 expression and inhibit the growth of PC cell lines under 2D culture conditions [24, 25, 29]. We therefore tested OGX‐427 delivery in 3D cultures of PC cell line‐derived spheroids and patient‐derived organoids (PDOs). PC‐3 cells were embedded in Matrigel at varying concentrations (35%, 50%, and 75%), grown for 3 days as spheroids and transfected in situ with FAM‐labeled HSP27 ASO (ASO‐FAM) using Oligofectamine (OF) (Figure 5A). ASO uptake in spheroids was more than 10‐fold lower than in 2D cultures transfected with OF and remained comparable to that of 2D cells incubated without transfection reagent (Figure 5B). Prolonging the incubation time to 96 h did not improve uptake (Figure 5B). Consistently, HSP27 knockdown was not achieved in OGX‐427‐transfected spheroids (Figure 5C), and no significant effect on spheroid size was observed (Figure 5D), indicating that functional delivery was also impaired.

**FIGURE 5:**
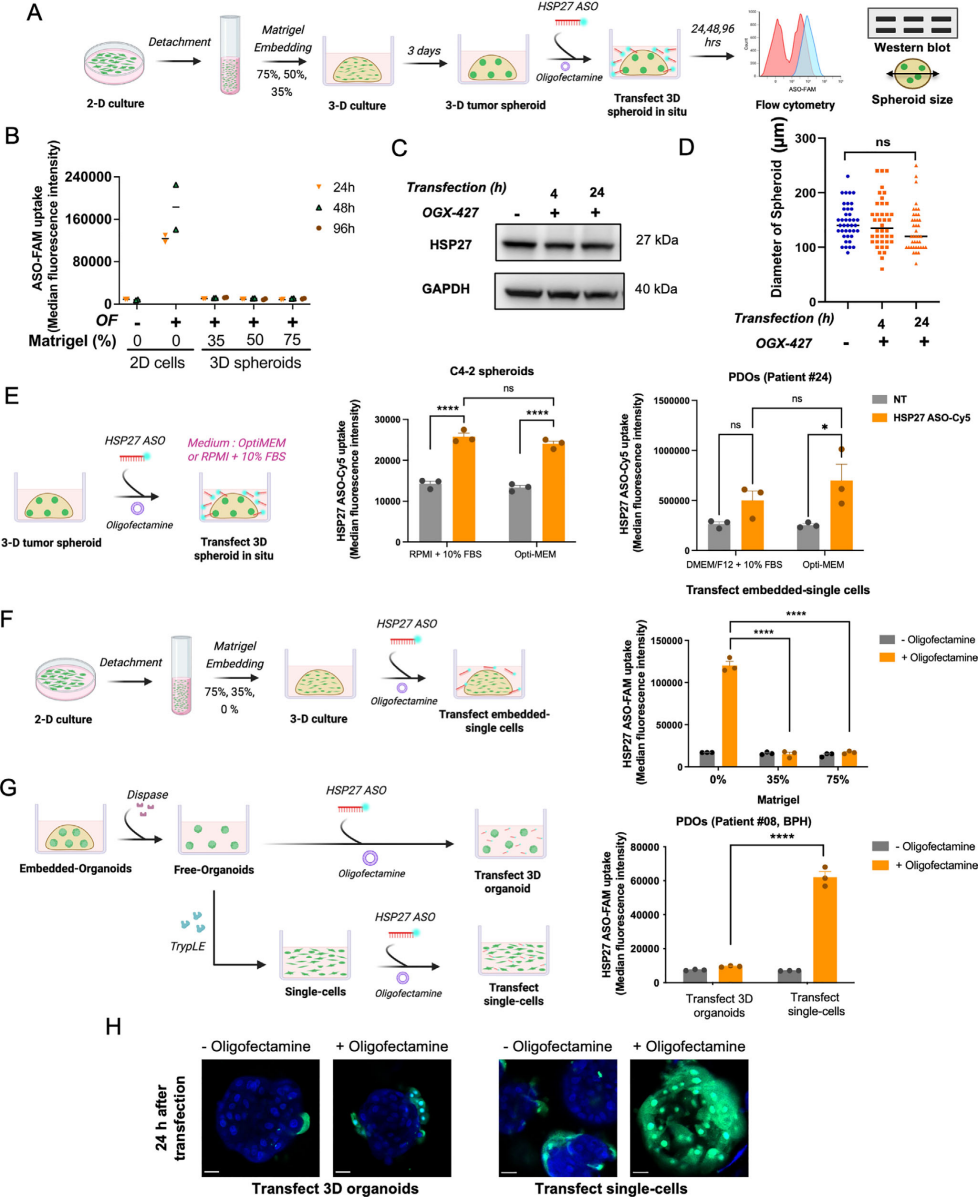
Matrigel and 3D architecture impair antisense oligonucleotide (ASO) delivery in prostate cancer spheroids and patient‐derived organoids (PDOs). (A) Experimental scheme describing the 3D spheroid in situ transfection of ASOs. PC‐3 cell‐derived spheroids embedded in varying concentrations of Matrigel (35%, 50%, and 75%) and transfected with FAM‐labeled ASO (ASO‐FAM) ± Oligofectamine; ASO uptake was measured by flow cytometry after 24, 48 or 96 h (B), and HSP27 expression and spheroid size were assessed after transfection with OGX‐427 (C, D). (B) Flow cytometry analysis and quantification of ASO‐FAM uptake in PC‐3 cell‐derived spheroids embedded in increasing concentrations of Matrigel (35%, 50%, and 75%) reveal markedly reduced uptake compared to 2D cultures, even after 96 h of incubation. Each symbol represents one well from a single experiment; no statistical test was applied to this panel. (C, D) Western blot analysis shows no detectable inhibition of HSP27 expression in Matrigel‐embedded PC‐3 spheroids transfected with OGX‐427 (150 nM) for 4 or 24 h (C), and no reduction in spheroid size (D), indicating poor functional delivery. (E) HSP27 ASO‐Cy5 uptake did not differ significantly between transfection in medium containing 10% FBS + Oligofectamine and in Opti‐MEM + Oligofectamine, in both C4‐2 3D spheroids (middle) and PDOs from Patient #24 (CSPC; right). NT, non‐treated. (F) PC‐3 cells were dissociated and re‐embedded in 35% or 75% Matrigel, followed by a 4‐h incubation with ASO‐FAM. Flow cytometry analysis of single cells within Matrigel (35% or 75%) demonstrated markedly reduced ASO uptake compared to Matrigel‐free controls, even when transfection reagent was applied. (G) Matrigel was digested with Dispase, and PDOs from Patient #08 (BPH) were either left intact or dissociated into single cells using TrypLE. Both formats were then treated with ASO‐FAM plus Oligofectamine for 4 h. ASO‐FAM uptake is significantly lower in intact 3D PDOs (without Matrigel) compared to dissociated single cells treated under identical conditions. (H) Confocal imaging shows ASO‐FAM signal (green) restricted to surface cells in intact PDOs, while reaggregated PDOs derived from single‐cell transfection show a widespread signal throughout the inner organoid structure. OF, Oligofectamine. Scale bars as indicated. Data are expressed as mean ± SEM (*n* = 3 technical replicates). Statistical significance was determined by two‐way ANOVA followed by Tukey's multiple comparisons test (E–G). A *p*‐value < 0.05 was considered statistically significant. *p < 0.05, **p < 0.01, ***p < 0.001, ****p < 0.0001. ns: nonsignificant. Schematic illustrations in this figure were created in BioRender.

Morgan et al. reported that preparing transfection complexes in culture medium supplemented with 10% FBS increased siRNA uptake into 3D structures [32]. We therefore compared this approach with our standard Opti‐MEM‐based protocol. In C4‐2 3D spheroid cultures, no significant difference was observed between ASO transfection using Opti‐MEM and complete medium (Figure 5E, middle panel). Moreover, the overall transfection efficiency in 3D cultures remained limited with both strategies. Similar results were confirmed in PDOs from Patient #24 cultured with 75% Matrigel (Figure 5E, right panel).

To study the potential impact of Matrigel on ASO delivery, PC‐3 cells were dissociated and re‐embedded in 35% or 75% Matrigel, then immediately incubated with ASO‐FAM for 4 h. Matrigel embedding abolished the Oligofectamin‐mediated increase in ASO uptake observed in Matrigel‐free cells (approximately 8‐fold reduction; Figure 5F). Direct incorporation of ASO‐FAM into Matrigel also failed to enhance delivery (data not shown), further supporting the inhibitory effect of the Matrigel matrix on ASO penetration.

To determine whether the 3D architecture itself further limits ASO delivery, we compared transfection of intact organoid with that of single cells in the absence of Matrigel. PDOs from Patient #08 (BPH) were released from Matrigel using Dispase, and either left intact or dissociated with TrypLE into single cells. Both formats were treated with ASO‐FAM plus Oligofectamine for 4 h in OptiMEM. ASO uptake in intact 3D PDOs remained low even in the absence of Matrigel, whereas dissociated single cells showed approximately 6‐fold higher uptake (Figure 5G). Confocal imaging 24 h after transfection showed that ASO‐FAM signals remained limited to the peripheral surface cells in intact organoids, whereas organoids reformed from transfected single cells displayed signal throughout their inner structure (Figure 5H).

Together, these results demonstrate that both Matrigel and the 3D architecture of spheroids and organoids constitute significant barriers to ASO delivery, while the single‐cell transfection protocol achieves the highest delivery efficacy.

### 2.4 OGX‐427 Inhibits the Growth of PC‐3 Spheroids and Prostate PDOs Following the Single‐Cell Transfection Protocol

Building on our findings that ASO uptake is significantly enhanced in single‐cell suspensions and in the absence of Matrigel, we applied this protocol to evaluate the therapeutic efficacy of OGX‐427 in both PC‐3‐derived spheroids and PDOs from multiple individuals in our cohort. Briefly, Matrigel was removed, and 3D structures were dissociated into single cells. Cells were then transfected with OGX‐427 using Oligofectamine in Opti‐MEM for 4 h at 37°C. Following transfection, cells were washed and resuspended in complete culture medium for 3D reformation.

At 72 h post‐transfection, OGX‐427 reduced PC‐3 spheroid viability by approximately 80% compared with SCR‐ASO (Figure 6A) and markedly suppressed PC‐3 spheroid formation (Figure 6B ), accompanied by a substantial reduction in HSP27 protein levels (Figure 6C). Importantly, OGX‐427 also reduced viability in a dose‐dependent manner in PDOs from all four patients tested, two with BPH (#08, #10) and two with CSPC (#16, #19) (Figure 6D), further supporting its broad activity. PDOs treated with control ASO reformed into organoids with normal morphology, whereas OGX‐427‐treated PDOs exhibited markedly smaller sizes and structural abnormalities (Figure 6E). Consistently, HSP27 protein levels were suppressed in OGX‐427‐treated PDOs, confirming effective on‐target molecular activity (Figure 6F).

**FIGURE 6:**
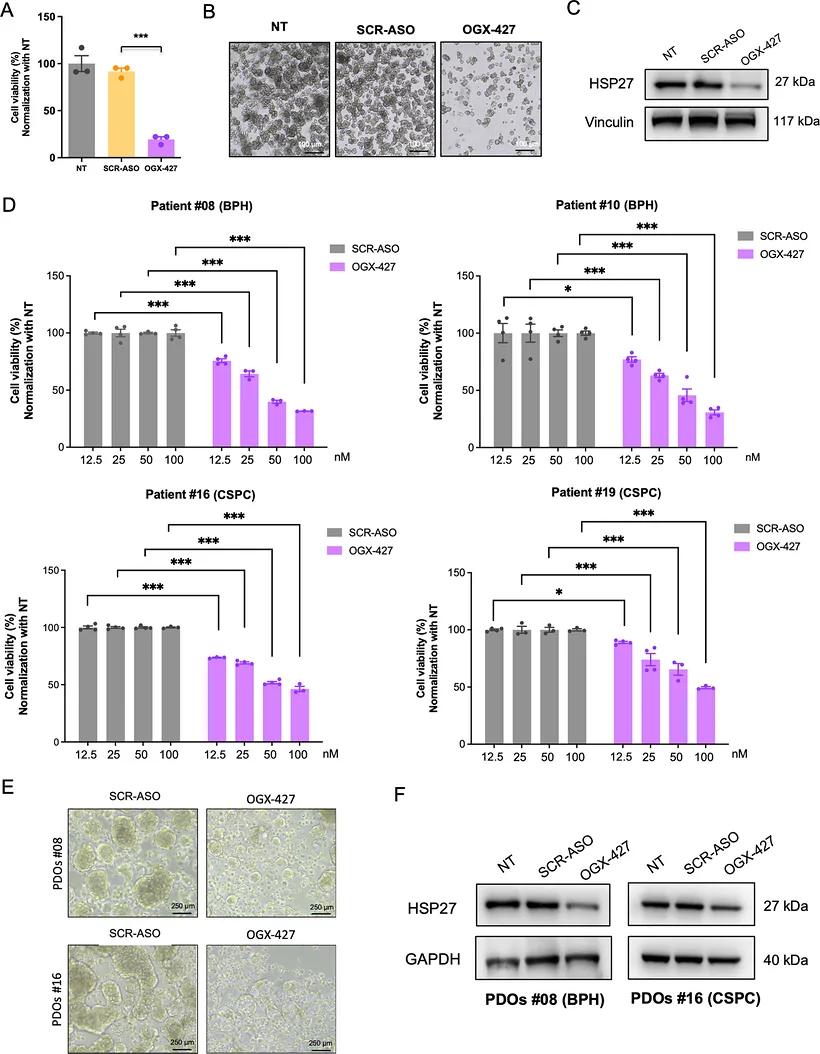
OGX‐427 inhibits growth and HSP27 expression in PC‐3 spheroids and prostate PDOs following Matrigel‐free single‐cell transfection. (A) Viability of PC‐3 spheroids 72 h after single‐cell transfection, normalized to non‐treated (NT) cells, was reduced by OGX‐427 (150 nM) compared to SCR‐ASO (150 nM). (B) Brightfield images show reduced formation and size of PC‐3‐derived spheroids 72 h after transfection with OGX‐427 compared to SCR‐ASO. Scale bars as indicated. (C) Western blot confirms downregulation of HSP27 protein in OGX‐427‐treated PC‐3 spheroids; vinculin, loading control. (D) Viability of PDOs from four patients (#08 and #10, BPH; #16 and #19, CSPC) 72 h after transfection with SCR‐ASO or OGX‐427 (12.5–100 nM), normalized to NT. (E) Representative brightfield images of PDOs from Patients #08 (BPH) and #16 (CSPC) show that OGX‐427 treatment leads to smaller, morphologically abnormal organoids compared to controls. (F) Western blot analysis of lysates from PDOs #08 and #16 demonstrates HSP27 suppression in OGX‐427‐treated samples, confirming effective on‐target activity. These data highlight the therapeutic potential of OGX‐427 when delivered through optimized transfection protocols in 3D prostate models. Data are expressed as mean ± SEM (*n* = 3 for A; *n* = 3–4 technical replicates per concentration for (D). Statistical significance was determined by two‐tailed Welch's *t*‐test (A; SCR‐ASO vs. OGX‐427, *P* = 0.0005) and, for each patient separately, by two‐way ANOVA followed by Bonferroni post‐tests comparing SCR‐ASO and OGX‐427 at each concentration (D). A *p*‐value < 0.05 was considered statistically significant. *p < 0.05, **p < 0.01, ***p < 0.001, ****p < 0.0001. ns: nonsignificant.

To further assess the applicability of the single‐cell transfection approach in PDOs, we evaluated two additional ASOs, ASO‐TCTP and ASO‐DDX5, which were previously identified in our laboratory as effective in reducing PC cell growth both in vitro and in xenograft models [26, 29]. IHC staining detected both DDX5 and TCTP in the parental tumors and matched organoids from Patient #24 (Figure 7A). Following Matrigel‐free single‐cell transfection, ASO‐TCTP and ASO‐DDX5 significantly reduced PDO viability in a dose‐dependent manner (Figure 7B), and increased the proportion of EthD‐1‐positive dead cells (Figure 7C). Both ASOs downregulated the expression of their respective target proteins, most clearly at 50 nM (Figure 7D). Collectively, these findings demonstrate the applicability of the single‐cell transfection strategy to different ASOs and highlight the potential of PDOs for evaluating RNA‐based therapies in a clinically relevant 3D setting.

**FIGURE 7:**
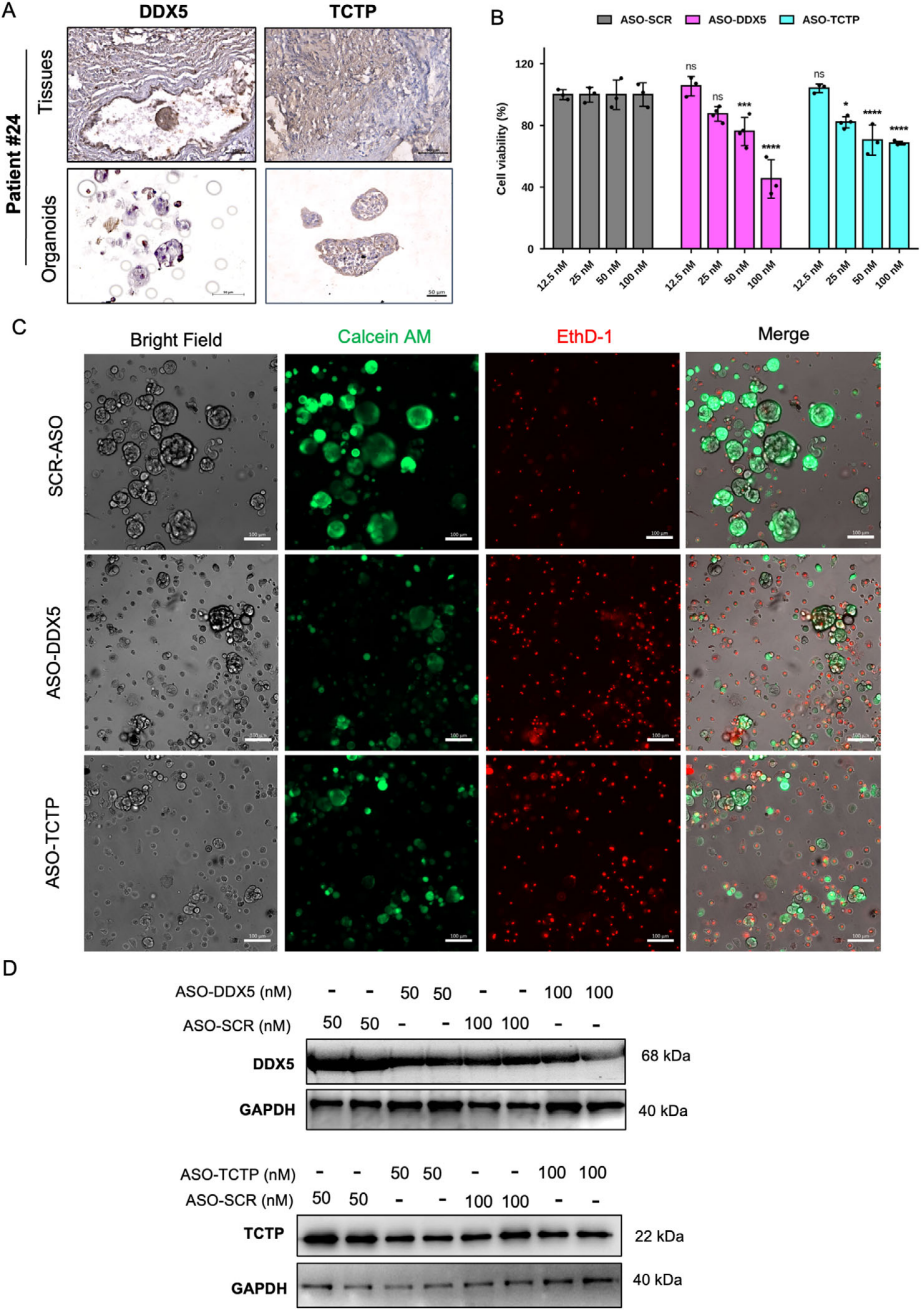
Effect of ASO‐TCTP and ASO‐DDX5 on the viability and target expression of prostate cancer (PC) 3D models following Matrigel‐free single‐cell transfection. (A) Immunohistochemistry of DDX5 and TCTP in the original tissue and PDOs from Patient #24 (CSPC; scale bars, 50 µm). (B) Cell viability assay shows reduced cell proliferation induced by ASO‐TCTP and ASO‐DDX5 compared to ASO‐SCR. (C) Representative brightfield and fluorescence images of organoids stained with Calcein AM (green, live cells) and ethidium homodimer‐1 (EthD‐1,red, dead cells) demonstrating organoid viability following treatment with ASO‐SCR, ASO‐DDX5 and ASO‐TCTP. (D) Western blot analysis of PDO lysates (duplicate lanes per condition) showed downregulation of DDX5 in ASO‐DDX5‐treated and TCTP in ASO‐TCTP‐treated samples compared with ASO‐SCR, confirming effective on‐target activity. Data are expressed as mean ± SD (*n* = 3–4 technical replicates per concentration). Statistical significance was determined by two‐way ANOVA followed by Dunnett's multiple comparisons test comparing ASO‐DDX5 and ASO‐TCTP with ASO‐SCR (control) at each concentration (B). A *p*‐value < 0.05 was considered statistically significant. *p < 0.05, **p < 0.01, ***p < 0.001, ****p < 0.0001. ns: nonsignificant.

## 3 Discussion

The aim of this study was to determine whether PC patient‐derived organoids (PDOs) can serve as functional avatars for antisense oligonucleotide (ASO)‐based personalized medicine, and to set up an efficient ASO therapeutic protocol in PC PDO models. In this work, we established a biobank of PC patient‐derived organoids (PDOs) from a cohort of 27 clinical specimens, including 12 benign prostatic hyperplasia (BPH), 14 castration‐sensitive prostate cancer (CSPC), and 1 castration‐resistant prostate cancer (CRPC) sample, most of which were collected via transurethral resection of the prostate (TURP). Applying a previously reported standardized protocol [18] allowed us to achieve an organoid formation success rate of over 90% (16/17, 94% for TURP specimens). This efficiency markedly exceeds previously reported rates for TURP specimens. For instance, Servant et al. documented a much lower success rate in generating organoids from TURP specimens, with only 4 out of 14 samples successfully cultured, compared with 55 out of 56 radical prostatectomy (RP) specimens [17]. RP is performed for localized PC, whereas TURP is the surgical “gold standard” for BPH and is also used as a palliative procedure in PC when local or metastatic tumor growth causes lower urinary tract obstruction, leading to recurrent gross hematuria, bladder clot retention, or subvesical obstruction that affects comfort, sleep, or daily activities [33]. TURP specimens therefore provide readily accessible tumor tissue from patients with locally advanced or metastatic disease, for whom RP is not indicated.

Our short‐term PDOs represent a promising preclinical model for precision medicine, particularly for RNA‐based therapeutics [6, 34]. Although none of our PDO lines were maintained in continuous culture beyond six months, most PC‐derived organoid lines (9 of 13) remained viable through at least two passages. Long‐term propagation of organoids from primary prostate tumors remains technically challenging, with most stable lines originating from metastatic lesions [13, 17]. This relatively limited propagation capacity is consistent with previous reports in PC organoid research and contrasts with tumor organoids derived from colorectal, gastric, or breast cancers, which can often be expanded long term. Indeed, primary prostate tumor epithelial cells are particularly difficult to sustain in vitro due to their strong dependence on androgen signaling, stromal interactions, as well as their susceptibility to early luminal differentiation and replicative senescence [13, 18]. In addition, prolonged passaging of prostate organoids has been associated with progressive overgrowth of benign epithelial populations and clonal drift, potentially compromising tumor fidelity [15, 17, 35]. Therefore, our study intentionally focused on early‐passage organoids to preserve the molecular and phenotypic characteristics of the original patient tumors and ensure maximal clinical relevance of the functional analyses.

Importantly, despite their limited long‐term expansion, our PDOs faithfully retained key histopathological and molecular characteristics of their parental tissues, including expression of basal (CK5) and luminal (CK8) cytokeratins, AR, PSMA, AMACR, STEAP1, PTEN, PSA, and HSP27. Moreover, the organoids derived from CSPC patients showed reduced growth in androgen‐depleted conditions, further confirming their functional relevance. However, androgen signaling was not systematically assessed in the remaining PDO samples, and its evaluation across a larger number of PDOs will be required to fully characterize the hormonal status of this biobank. Collectively, these findings highlight the value of short‐term PDO cultures as a clinically relevant platform for preclinical drug testing, particularly in the early stages of disease.

Organoids can serve as reliable models for drug discovery across various cancer types, including PC. Most studies to date have focused successfully on small‐molecule drugs, such as enzalutamide (androgen receptor inhibitor), abiraterone (CYP17 inhibitor), everolimus (mTOR inhibitor), and BKM120 (PI3K inhibitor) [13, 17]. Only a few studies have attempted to test ASOs in organoid systems, and none, to our knowledge, has addressed ASO delivery barriers in PC organoids [36, 37, 38]. A major obstacle in this context is poor drug penetration due to the dense extracellular matrix [11, 12, 39] and the 3D architecture of organoids. While our team observed good efficacy of small molecules in Matrigel‐embedded 3D organoids [20], the efficacy of OGX‐427 ASO was severely limited under these conditions. We showed that both Matrigel and the 3D structure impede ASO delivery, as evidenced by poor uptake and minimal gene knockdown. By optimizing ASO delivery through Matrigel removal and single‐cell transfection, we achieved effective target gene inhibition and growth suppression in prostate PDO models. To our knowledge, this is the first detailed report demonstrating the impact of 3D architecture on ASO delivery in PC organoids and proposing a practical strategy to overcome it, thereby enabling the use of PDOs to assess ASO‐based personalized therapy.

Importantly, the translational relevance of our PDO platform for RNA‐based precision medicine was further supported by our recent study investigating combined targeting of HSP27 and mTOR signaling in advanced prostate cancer models [19]. Using the same single‐cell transfection strategy, we evaluated OGX‐427 in combination with sapanisertib, an mTOR inhibitor, in PDOs established from several PC patients exhibiting high HSP27 expression and activated mTOR signaling. This combinatorial approach induced a strong synergistic anti‐tumor effect in PDOs, consistent with observations obtained in CRPC cell lines and xenograft models, thereby validating the predictive and translational potential of our organoid platform across multiple preclinical systems. These findings further emphasize the value of short‐term PDO cultures as clinically relevant tools for the development and functional evaluation of ASO‐based therapeutic strategies in PC.

While organoid dissociation is required to achieve efficient intracellular delivery of ASOs, the resulting single cells readily reconstituted 3D organoids after transfection, thereby preserving the opportunity to evaluate ASO efficacy within a physiologically relevant multicellular structure. Thus, this approach provides a practical platform for evaluating ASO activity in patient‐derived tumor cells. Ongoing studies explore alternative delivery strategies to improve ASO uptake in intact organoids and facilitate clinical translation. Advances in ASO chemistry, including phosphorothioate backbones, 2′‐*O*‐methoxyethyl (2′‐MOE), locked nucleic acid (LNA), and constrained ethyl (cEt) modifications, have been shown to enhance nuclease resistance, target affinity, and cellular uptake, potentially reducing the need for transfection reagents [40]. In parallel, nanoparticle‐based delivery systems, such as lipid nanoparticles, liposomes, and lipid‐modified ASOs, can improve ASO stability, endosomal escape, and tissue penetration [7, 41, 42]. Targeted nanocarriers, including antibody‐functionalized liposomes and lipid–oligonucleotide conjugates, have demonstrated enhanced tumor delivery and therapeutic efficacy of oligonucleotide drugs in preclinical cancer models [43]. Additional approaches, such as conjugation of ASOs to targeting ligands or cell‐penetrating peptides, as well as the use of permeability‐enhancing or matrix‐modifying agents, may further improve diffusion and uptake within intact 3D tumor tissues [44]. Calcium‐enhanced delivery improves membrane permeability and facilitates uptake through a simple medium modification, but its efficiency can be variable and high calcium exposure may influence intracellular signaling pathways [45]. Magnetic‐guided delivery, which employs nanoparticle‐ASO complexes and external magnetic fields to promote deeper penetration in 3D cultures, offers spatial control but requires specialized materials and raises concerns regarding nanoparticle safety and biocompatibility [46]. Collectively, these strategies offer promising avenues to overcome current delivery limitations and enhance the translational potential of ASO‐based therapies for PC.

In conclusion, we successfully established a biobank of prostate PDOs, mainly from TURP specimens, achieving a high initial organoid establishment success rate (>90%) while preserving the histological architecture and molecular characteristics of the original tumors. Using this platform, we identified the extracellular matrix and 3D organoid architecture as major barriers to ASO delivery and therapeutic efficacy. By removing Matrigel and performing transfection at the single‐cell stage, we achieved efficient ASO uptake, robust target gene silencing, and significant inhibition of organoid growth. These findings highlight the importance of optimizing delivery strategies for nucleic acid therapeutics in complex 3D tumor models and establish a practical framework for evaluating ASO‐based therapies in PDOs. Future studies will focus on improving long‐term organoid culture, expanding the biobank to capture greater tumor heterogeneity, and further leveraging this platform to accelerate the development of precision medicine approaches and RNA‐targeted therapeutics for PC and other solid malignancies.

## 4 Experimental Section

### 4.1 Collection and Establishment of PC Patient‐Derived Organoids

A total of 27 fresh prostate tissue samples were collected by Dr Michael Baboudjian from patients who underwent transurethral resection of the prostate (TURP) and biopsy procedures performed at North Hospital (AP‐HM), Marseille, France, following AP‐HM Biological Resources Center Authorization (#AC‐2023‐5572 and #AC‐2024‐6402). Written informed consent was obtained from all patients prior to sample collection, and all procedures were performed in accordance with the Declaration of Helsinki (see Ethics Approval and Consent to Participate). Samples were confirmed as benign or tumorous, with Gleason scores assigned based on hematoxylin eosin saffron (HES) staining by pathologists. Detailed clinical and pathological characteristics of patient samples are described in Table S1. Immediately after resection, fresh tissue samples were placed in cold advanced DMEM/F12 +/+/+ (containing penicillin/streptomycin, GlutaMAX 100× diluted and 10 mM Hepes) (all reagents from Thermo Fisher Scientific, Life Technologies SAS, Villebon‐sur‐Yvette, France) and transported on ice to the laboratory. The time between resection and tumor cell isolation was kept under 5 h. For each sample, the majority of tissue fragments were used for organoid culture, while the smaller portions were allocated for protein extraction or fixed in 4% paraformaldehyde for histological analysis.

The isolation of tumor cells from prostate tissue resection and organoid culture was performed as described by Drost et al. [18], using overnight digestion in 5 mg/mL collagenase II with 10 µM Y‐27632 for all specimens, including tumor tissues and biopsies. Prostate tissues (2–5 mm) were cut into fine pieces (<2 mm^3^) using a sterile scalpel, washed with cold adDMEM/F12 +/+/+, and digested in 5–10 mL of 5 mg/ml collagenase type II (17101‐015, Gibco, Thermo Fisher Scientific, Life Technologies SAS, Villebon‐sur‐Yvette, France) in adDMEM/F12 +/+/+ supplemented with rho‐associated coiled‐coil containing protein kinase (ROCK) inhibitor Y‐27632 at 10 µM overnight at 37°C on a rocking platform. The next day, the cell and aggregate suspension was digested into single cells using TrypLE (12605010, Gibco, Thermo Fisher Scientific, Life Technologies SAS, Villebon‐sur‐Yvette, France) for about 15–20 min at 37°C. The mixture was pipetted up and down every 5 min. After incubation, adDMEM/F12 +/+/+ was added, and the cell mixture was filtered through a 70 µm cell strainer to remove debris. The cells were counted and frozen to create a storage bank of primary prostate cells. A portion of cells was mixed with 75% Matrigel (Corning, 354253) and plated as droplets of 25 000 cells/40 µL and cultured in the complete growth factor medium. The medium was changed twice weekly. Depending on the sample, the organoids were cultured for one to three weeks before passaging. The organoid medium composition is listed in Table S4.

### 4.2 Passage of Organoids, Estimating Organoid Formation Efficiency and Measuring Diameter

Organoids embedded in Matrigel were first incubated with Dispase 1 U/mL (STEMCELL Technologies, 07923) for 1 h at 37°C. To break the Matrigel dome, the suspension was pipetted up and down several times using a 1 mL pipette tip pre‐wetted with Dispase. After 1 h of incubation, organoids were washed once with adDMEM/F12 +/+/+ and centrifuged at 300 × g for 5 min to remove the Matrigel. The organoids were further digested by incubating with 2–3 mL of TrypLE at 37°C for 15 min, with up‐and‐down pipetting every 5 min. The cell suspension was washed with adDMEM/F12 +/+/+ and cells were counted and seeded as 40 µL droplets containing 25 000 cells in 75% Matrigel.

To quantify organoids, a 10 µL aliquot of the 1 mL medium containing the Dispase‐released organoids was counted under a microscope. Organoid formation efficiency (OFE) is defined as: high OFE with more than 100 organoids/dome (+++), medium OFE with 50–100 organoids/dome (++), and low OFE with fewer than 50 organoids/dome (+). For diameter measurements, 77–115 organoids per patient were randomly selected and measured using ImageJ software.

### 4.3 Cell Lines and PC Cell Line‐Derived Spheroids

The human PC cell lines C4‐2 (RRID:CVCL_4782) and PC‐3 (RRID:CVCL_0035) were obtained from the American Type Culture Collection (ATCC, Manassas, VA, USA) in 2012. Cell line authentication was performed by the supplier, and cells were tested monthly for mycoplasma contamination. Cells were dissociated into single cells using TrypLE, counted, and seeded in 40 µL Matrigel droplets at 5000 cells per droplet. The cells and spheroids were cultured in RPMI‐1640 medium (Gibco, Thermo Fisher Scientific, Life Technologies SAS, Villebon‐sur‐Yvette, France) supplemented with 10% fetal bovine serum at 37°C in 5% CO_2_.

### 4.4 Histology, Immunohistochemistry, and Immunofluorescence

For all biomolecular analyses, Matrigel was removed from organoids by incubating them with 1 U/mL Dispase II (07923, STEMCELL Technologies) for 60 min. Organoids were then fixed in 4% PFA for 1 h. Histogel (Thermo Fisher Scientific, Life Technologies SAS, Villebon‐sur‐Yvette, France, HG4000‐012) was used to surround the organoids before paraffin embedding as described by Fujii et al. [47]. Hematoxylin‐eosin‐saffron (HES) staining was performed at the Pathological Anatomy Laboratory, AP‐HM La Timone, Marseille, France.

For immunohistochemistry (IHC) and immunofluorescence (IF), organoid and tissue sections were deparaffinized and washed with phosphate‐buffered saline (PBS). Antigen retrieval was performed in citrate buffer (Agilent, S169984‐2) for 20 min at 95°C. Sections were incubated in blocking solution (5% BSA, 0.2% Triton X‐100, 0.05% Tween‐20 in PBS) for 1 h, followed by overnight incubation at 4°C with primary antibodies diluted in antibody diluent (Dako antibody diluent). The next day, IHC sections were washed, and endogenous peroxidase activity was quenched with 3% hydrogen peroxide for 10 min. The biotinylated secondary antibodies were incubated for 2 h at room temperature before developing the signal using VECTASTAIN ABC systems and ImmPACT DAB peroxidase substrate (Vector Laboratories, PK‐4000, SK‐4105) for 2–10 min. The IHC sections were counterstained with hematoxylin, dehydrated, and mounted before imaging using an Axioscan Z1 slide scanner (Zeiss). Details of all antibodies and dilutions are provided in Table S3.

For IF analysis, following primary antibody incubation, sections were incubated with fluorophore‐conjugated secondary antibodies. Sections were then mounted using Fluorogel mounting medium containing DAPI. Images were captured using a Zeiss Axio Imager Z2 Apotome.2 or Zeiss LSM 800 confocal microscope.

### 4.5 RNA Isolation and RT‐qPCR

Organoids were stored at ‐80°C. For total RNA isolation, the organoids were first homogenized in Fisherbrand Pre‐Filled Bead Mill Tubes containing ceramic beads (Fisher Scientific) and filled with 0.4 mL RLT lysis buffer (Qiagen GmbH) using FastPrep‐24 Classic. The lysate was mixed with 0.4 mL TRIzol reagent, followed by 0.2 mL chloroform, then centrifuged at 10 000 x g for 15 min at 4°C. The upper aqueous phase containing total RNA was mixed with an equal volume of 70% ethanol. RNA purification was performed using RNeasy Mini spin columns according to the manufacturer's protocol. RNA integrity was evaluated using a Bioanalyzer 2100 (Agilent Technologies). RNA Integrity Numbers (RIN) between 8 and 10 were accepted for cDNA synthesis. Total RNA was used for cDNA synthesis with SuperScript VILO MasterMix (Invitrogen, Thermo Fisher Scientific, Life Technologies SAS, Villebon‐sur‐Yvette, France). qPCR reactions were performed using the TaqMan Universal PCR Master Mix (Applied Biosystems, Thermo Fisher Scientific, Life Technologies SAS, Villebon‐sur‐Yvette, France) and run on StepOnePlus Real‐Time PCR System (Applied Biosystems). The expression level of HSP27 was used for subsequent statistical analysis. HSP27 expression was normalized to GAPDH. The sequences of primers and probes are listed in Table S5.

### 4.6 Mutational Analysis by PCR and Sanger Sequencing

Genomic DNA was extracted from patient‐derived organoids (PDOs) using standard extraction procedures from the DNeasy Blood & Tissue kit (Qiagen). Specific genomic regions containing reported tumor‐associated mutations were amplified by PCR using gene‐specific primers designed to flank the mutation sites (Table S5). PCR amplification was performed under standard cycling conditions optimized for each target region using GoTaq Colorless Master Mix (Promega). Amplified products were verified by agarose gel electrophoresis to confirm correct amplicon size and specificity. PCR products were then purified using a PCR purification kit (Qiagen) according to the manufacturer's instructions. Purified amplicons were subsequently submitted for Sanger sequencing (Microsynth). Sequencing chromatograms were analyzed using FinchTV chromatogram viewer and compared to the corresponding reference sequences to determine the presence or absence of reported mutations.

### 4.7 Protein Extraction and Western Blot Analysis

PC cell line‐derived spheroids and PDOs were incubated with Dispase and washed twice in cold DPBS to remove Matrigel. Samples were homogenized in Fisherbrand Pre‐Filled Bead Mill Tubes containing ceramic beads (Fisher Scientific) and lysed in lysis buffer containing protease and phosphatase inhibitors (Thermo Fisher Scientific, Life Technologies SAS, Villebon‐sur‐Yvette, France, A32965, A32957). Protein was collected in low‐binding protein tubes. Protein concentration was determined using BCA assay (Thermo Fisher Scientific, Life Technologies SAS, Villebon‐sur‐Yvette, France, 23225). A total of 25 µg of protein was loaded onto 4%–12% gradient polyacrylamide gels (Invitrogen, Thermo Fisher Scientific, Life Technologies SAS, Villebon‐sur‐Yvette, France, NW04122BOX) and transferred to a PVDF membrane. Membranes were incubated with antibodies as listed in Table S3. The protein bands were detected using the enhanced chemiluminescence reagent (ECL Prime Western Blotting System, Cytiva, RPN2232), and signal analysis was performed with ImageJ software (NIH, Bethesda, MD, USA).

### 4.8 Antisense Oligonucleotide (ASO) Delivery in 3D Prostate Cancer Models

Cells at 50% confluence or Matrigel‐embedded samples were washed once with D‐PBS and incubated with FAM‐labeled ASO (ASO‐FAM) at 250 nM in Opti‐MEM medium ± Oligofectamine (4 µL/mL) (Thermo Fisher Scientific, Life Technologies SAS, Villebon‐sur‐Yvette, France) for 4 h. The procedure was repeated up to four times over 4 days, depending on the experiment. For HSP27 knockdown in Matrigel‐embedded spheroids, OGX‐427 was used at 150 nM under the same conditions. Samples were then incubated with Dispase, followed by TrypLE to obtain single cells. Cells were washed once before analyzing ASO‐FAM signal intensity on CytoFlex S (Beckman Coulter, Inc.).

Alternatively, 3D structures were dissociated into single cells and transfected with ASO‐FAM, OGX‐427, or ASO‐control ± Oligofectamine at different concentrations in Opti‐MEM for 4 h in Cell‐Repellent plates (Greiner Bio‐One system, 662970). After incubation, the ASO and Oligofectamine mixture was removed. Cells were either used for ASO delivery measurement or resuspended in complete medium for 72 h before phenotype and biomolecular evaluation.

### 4.9 Viability Analysis

Cells were seeded at 5000 cells per well in 96‐well plates under two conditions: with or without Matrigel. For the condition without Matrigel, cells were seeded in ultra‐low adherence plates (Greiner Bio‐One system, 655970). For the condition with Matrigel, cells were embedded in 10 µL of 75% Matrigel. For drug treatments, organoids were allowed to grow for 2 weeks and were treated with vehicle (DMSO) control or enzalutamide (NB‐64‐30476, CliniScience) (0.5–10 µM), or olaparib (HY‐10162, CliniScience) or abiraterone (HY‐70013, CliniScience) (0.5–10 µM) for 2 days. Cell viability was assessed using the CellTiter‐Glo 3D luminescence assay (Promega, G9682) according to the manufacturer's guidelines. Live and dead cells within organoids were assessed using the LIVE/DEAD Viability/Cytotoxicity Kit (Calcein AM/EthD‐1, Molecular Probes, Thermo Fisher Scientific). Calcein AM is converted by intracellular esterases into fluorescent calcein in viable cells (green fluorescence), whereas EthD‐1 enters cells with compromised plasma membranes and stains nucleic acids in dead cells (red fluorescence). Stained organoids were imaged using fluorescence microscopy. All viability (drug) treatment experiments were performed in triplicate.

### 4.10 Statistical Analysis

Statistical analyses were performed using GraphPad Prism 10 software (GraphPad Software, San Diego, CA, USA). Cell viability data were normalized to the corresponding untreated (NT), vehicle, or control condition (set as 100%). ASO uptake was quantified by flow cytometry as the median fluorescence intensity (MFI). Western blot band intensities were quantified using ImageJ and normalized to the corresponding loading control (GAPDH or vinculin), and relative mRNA expression was calculated using the 2^−ΔΔCt^ method. No data points were excluded as outliers. Data are presented as mean ± standard error of the mean (SEM), except for Figure 7, in which data are presented as mean ± standard deviation (SD). The sample size (*n*) for each analysis, and whether it corresponds to technical replicates or independent experiments, is indicated in the figure legends. Comparisons between two groups were performed using a two‐tailed unpaired *t*‐test with Welch's correction. Comparisons among three or more groups were performed using ordinary one‐way or two‐way ANOVA, followed by Dunnett's test (comparisons with a control group), Tukey's test (comparisons between selected groups), or Bonferroni's test (comparison of two treatments at each concentration) to correct for multiple comparisons, as indicated in the figure legends. A two‐sided adjusted *P* value < 0.05 was considered statistically significant (*P < 0.05, **P < 0.01, ***P < 0.001, ****P < 0.0001).

## Author Contributions

P.R., T.K.L. supervised the study; T.T.T. led and performed most of the experiments; T.T.U.P., Q.H.D., T.K.L. helped conduct the experiments and analyzed data; M.B. collected samples and clinico‐pathologic data; S.G., L.D. performed the pathological assessment; I.S. performed ASO synthesis; T.T.L. designed primers, performed PCR and supported mutation analysis; P.B., B.G. supported the IHC experiments; V.B. discussed the project; C.B. discussed the project, specifically the assessment of human samples and the pathological assessment; T.K.L., T.T.T., T.T.U.P. wrote the original draft manuscript, and prepared the figures; P.R., T.K.L., D.T., C.G. critically reviewed and edited the manuscript. P.R., D.T., T.K.L. obtained the project grants. The manuscript has been read and approved by all named authors.

## Funding

This work was supported by INSERM and grants from the Amidex Fondation “Interdisciplinarity” project (mRNAnanoPSMA), the Ligue Nationale Contre Le Cancer (R22030AA), ITMO cancer (C21033AS), the Fondation de France (00131140/WB‐2022‐44423) and Amidex Excellence Initiative, Imaging Institute (AMX‐19‐IET‐002). The PhD studies of T.T.U.P. and Q.H.D. are funded by the French Excellence Scholarship. T.K.L.’s postdoc fellowship was funded by the ITMO Cancer (C21033AS).

## Ethics Statement

Prostate tissue samples from all patients included in this study (PC and BPH) were collected during routine clinical care, without deviation from standard guidelines. Written informed consent was obtained from all participants and recorded in their medical records, and the patients were also informed that their samples could be used for research purposes. All procedures were performed in accordance with the Declarations of Helsinki and Istanbul. The sample collection was declared to the French Ministry of Research (CODECOH) through the Biological Resource Centre (BRC) of the Marseille Public University Hospital System (AP‐HM; identification number: 0CPREP01E001). The samples used in this study were provided by the AP‐HM BRC (authorization #AC‐2023‐5572 and #AC‐2024‐6402). The BRC has been certified according to ISO 9001 since 8 December 2023, for activities conducted in accordance with ISO 20387, and is authorized by the French Ministry of Higher Education, Research and Innovation to supply biological resources for research. Clinical data were collected prospectively and anonymously in a dedicated database accessible only to authorized investigators.

## Conflicts of Interest

P.R. is a cofounder and stakeholder of SILON Therapeutics (https://silontx.com), a biotech company focused on precision medicine and nucleic acid therapeutics. D.T. is a cofounder and stakeholder of SILON Therapeutics and reports receiving advisory board honoraria from Novartis. The University of British Columbia has filed patent applications for OGX‐427 (apatorsen), an antisense inhibitor of HSP27, with P.R. listed as an inventor. This intellectual property has been licensed to OncoGenex Technologies, a biotechnology company headquartered in Vancouver, Canada.

## Supporting information




**Supporting File**: adhm71822‐sup‐0001‐SuppMat.docx.

## Data Availability

The data that support the findings of this study are available in the supplementary material of this article.

## References

[1] F. Bray , M. Laversanne , H. Sung , et al., “Global Cancer Statistics 2022: GLOBOCAN Estimates of Incidence and Mortality Worldwide for 36 Cancers in 185 Countries,” CA: A Cancer Journal for Clinicians 74 (2024): 229. 10.3322/caac.21834 38572751

[2] D. Tilki , R. C. N. van den Bergh , E. Briers , et al., “EAU‐EANM‐ESTRO‐ESUR‐ISUP‐SIOG Guidelines on Prostate Cancer. Part II—2024 Update: Treatment of Relapsing and Metastatic Prostate Cancer,” European Urology 86 (2024): 164–182. 10.1016/j.eururo.2024.04.010 38688773

[3] I. B. Riaz , S. A. A. Naqvi , H. He , et al., “First‐Line Systemic Treatment Options for Metastatic Castration‐Sensitive Prostate Cancer,” JAMA Oncology 9 (2023): 635. 10.1001/jamaoncol.2022.7762 PMC9982744 36862387

[4] T. K. Le , Q. H. Duong , V. Baylot , et al., “Castration‐Resistant Prostate Cancer: From Uncovered Resistance Mechanisms to Current Treatments,” Cancers 15 (2023): 5047. 10.3390/cancers15205047 PMC10605314 37894414

[5] V. Baylot , T. K. Le , D. Taïeb , P. Rocchi , and L. Colleaux , “Between Hope and Reality: Treatment of Genetic Diseases Through Nucleic Acid‐Based Drugs,” Communications Biology 7 (2024): 489. 10.1038/s42003-024-06121-9 PMC11039704 38653753

[6] S. Karkampouna , F. La Manna , A. Benjak , et al., “Patient‐Derived Xenografts and Organoids Model Therapy Response in Prostate Cancer,” Nature Communications 12 (2021): 1117. 10.1038/s41467-021-21300-6 PMC7892572 33602919

[7] Q. H. Duong , G. Oliver , T. K. Le , et al., “HSP27 mRNA Silencing and Radiation‐Driven Endosomal‐Lysosomal Escape of Lipid Nanomicelles Synergistically Radiosensitize Castration‐Resistant Prostate Cancer Cells,” OpenNano 29 (2026): 100300.

[8] S. Udu‐Ituma , J. Adélaïde , T. K. Le , et al., “ZNF703 mRNA‐Targeting Antisense Oligonucleotide Blocks Cell Proliferation and Induces Apoptosis in Breast Cancer Cell Lines,” Pharmaceutics 15 (2023): 1930. 10.3390/pharmaceutics15071930 PMC10384502 37514116

[9] T. K. Le , C. Paris , K. S. Khan , F. Robson , W.‐L. Ng , and P. Rocchi , “Nucleic Acid‐Based Technologies Targeting Coronaviruses,” Trends in Biochemical Sciences 46 (2021): 351–365. 10.1016/j.tibs.2020.11.010 PMC7691141 33309323

[10] G. Vlachogiannis , S. Hedayat , A. Vatsiou , et al., “Patient‐Derived Organoids Model Treatment Response of Metastatic Gastrointestinal Cancers,” Science 359 (2018): 920. 10.1126/science.aao2774 PMC6112415 29472484

[11] H. Yang , L. Sun , M. Liu , and Y. Mao , “Patient‐Derived Organoids: A Promising Model for Personalized Cancer Treatment,” Gastroenterology Report 6 (2018): 243–245. 10.1093/gastro/goy040 PMC6225812 30430011

[12] Z. Zhou , L. Cong , and X. Cong , “Patient‐Derived Organoids in Precision Medicine: Drug Screening, Organoid‐on‐a‐Chip and Living Organoid Biobank,” Frontiers in Oncology 11 (2021): 762184. 10.3389/fonc.2021.762184 PMC8755639 35036354

[13] D. Gao , I. Vela , A. Sboner , et al., “Organoid Cultures Derived From Patients With Advanced Prostate Cancer,” Cell 159 (2014): 176. 10.1016/j.cell.2014.08.016 PMC4237931 25201530

[14] V. Rago , A. Perri , and S. Di Agostino , “New Therapeutic Perspectives in Prostate Cancer: Patient‐Derived Organoids and Patient‐Derived Xenograft Models in Precision Medicine,” Biomedicines 11 (2023): 2743. 10.3390/biomedicines11102743 PMC10604340 37893116

[15] M. Beshiri , S. Agarwal , J. J. Yin , and K. Kelly , “Prostate Organoids: Emerging Experimental Tools for Translational Research,” Journal of Clinical Investigation 133 (2023): 169616. 10.1172/JCI169616 PMC10178834 37183816

[16] L. Puca , R. Bareja , D. Prandi , et al., “Patient Derived Organoids to Model Rare Prostate Cancer Phenotypes,” Nature Communications 9 (2018): 2404. 10.1038/s41467-018-04495-z PMC6008438 29921838

[17] R. Servant , M. Garioni , T. Vlajnic , et al., “Prostate Cancer Patient‐Derived Organoids: Detailed Outcome From a Prospective Cohort of 81 Clinical Specimens,” Journal of Pathology 254 (2021): 543–555. 10.1002/path.5698 PMC8361965 33934365

[18] J. Drost , W. R. Karthaus , D. Gao , et al., “Organoid Culture Systems for Prostate Epithelial and Cancer Tissue,” Nature Protocols 11 (2016): 347–358. 10.1038/nprot.2016.006 PMC4793718 26797458

[19] Q. H. Duong , T. K. Le , T. T. Tran , et al., “HSP27 Functional Switch Drives Castration‐Resistant Prostate Cancer via mTOR Pathway Activation, Highlighting Promising Combination Therapies,” Journal of Experimental & Clinical Cancer Research 45 (2026): 98. 10.1186/s13046-026-03695-6 PMC13085302 41877198

[20] Q. H. Duong , E. Khusnutdinova , T. K. Le , et al., “A Novel 5‐FU Derivative Chemotherapy: A Promising Pan‐Cancer Treatment to Improve Antitumor Efficacy and Overcome Therapy Resistance,” Journal of Experimental & Clinical Cancer Research (2026), 10.1186/s13046-026-03826-z.

[21] E. Y. Yu , S. L. Ellard , S. J. Hotte , et al., “A Randomized Phase 2 Study of a HSP27 Targeting Antisense, apatorsen With Prednisone Versus Prednisone Alone, in Patients With Metastatic Castration Resistant Prostate Cancer,” Investigational New Drugs 36 (2018): 278–287. 10.1007/s10637-017-0553-x 29250742

[22] J. E. Rosenberg , N. M. Hahn , M. M. Regan , et al., “Apatorsen Plus Docetaxel Versus Docetaxel Alone in Platinum‐Resistant Metastatic Urothelial Carcinoma (Borealis‐2),” British Journal of Cancer 118 (2018): 1434–1441. 10.1038/s41416-018-0087-9 PMC5988804 29765151

[23] P. Rocchi , A. So , S. Kojima , et al., “Heat Shock Protein 27 Increases After Androgen Ablation and Plays a Cytoprotective Role in Hormone‐Refractory Prostate Cancer,” Cancer Research 64 (2004): 6595–6602. 10.1158/0008-5472.CAN-03-3998 15374973

[24] P. Rocchi , E. Beraldi , S. Ettinger , et al., “Increased Hsp27 After Androgen Ablation Facilitates Androgen‐Independent Progression in Prostate Cancer via Signal Transducers and Activators of Transcription 3–Mediated Suppression of Apoptosis,” Cancer Research 65 (2005): 11083–11093. 10.1158/0008-5472.CAN-05-1840 16322258

[25] C. Andrieu , D. Taieb , V. Baylot , et al., “Heat Shock Protein 27 Confers Resistance to Androgen Ablation and Chemotherapy in Prostate Cancer Cells Through eIF4E,” Oncogene 29 (2010): 1883–1896. 10.1038/onc.2009.479 20101233

[26] V. Baylot , M. Katsogiannou , C. Andrieu , et al., “Targeting TCTP as a New Therapeutic Strategy in Castration‐Resistant Prostate Cancer,” Molecular Therapy 20 (2012): 2244–2256. 10.1038/mt.2012.155 PMC3519986 22893039

[27] H. Ziouziou , C. Andrieu , E. Laurini , et al., “Targeting Hsp27/eIF4E Interaction With Phenazine Compound: A Promising Alternative for Castration‐Resistant Prostate Cancer Treatment,” Oncotarget 8 (2017): 77317–77329. 10.18632/oncotarget.20469 PMC5652782 29100389

[28] C. Cherif , D. T. Nguyen , C. Paris , et al., “Menin Inhibition Suppresses Castration‐Resistant Prostate Cancer and Enhances Chemosensitivity,” Oncogene 41 (2022): 125–137. 10.1038/s41388-021-02039-2 PMC8724010 34711954

[29] T. K. Le , C. Cherif , K. Omabe , et al., “DDX5 mRNA‐targeting Antisense Oligonucleotide as a New Promising Therapeutic in Combating Castration‐Resistant Prostate Cancer,” Molecular Therapy 31 (2023): 471–486. 10.1016/j.ymthe.2022.08.005 PMC9931527 35965411

[30] A. Bourefis , H. Berredjem , O. Djeffal , T. K. Le , S. Giusiano , and P. Rocchi , “HSP27/Menin Expression as New Prognostic Serum Biomarkers of Prostate Cancer Aggressiveness Independent of PSA,” Cancers 14 (2022): 4773. 10.3390/cancers14194773 PMC9562023 36230697

[31] H. Ziouziou , C. Paris , S. Benizri , et al., “Nucleoside‐Lipid‐Based Nanoparticles for Phenazine Delivery: A New Therapeutic Strategy to Disrupt Hsp27‐eIF4E Interaction in Castration Resistant Prostate Cancer,” Pharmaceutics 13 (2021): 623. 10.3390/pharmaceutics13050623 PMC8146835 33925528

[32] R. G. Morgan , A. C. Chambers , D. N. Legge , S. J. Coles , A. Greenhough , and A. C. Williams , “Optimized Delivery of siRNA Into 3D Tumor Spheroid Cultures In Situ,” Scientific Reports 8 (2018): 7952. 10.1038/s41598-018-26253-3 PMC5962539 29785035

[33] A. Heidenreich , C. Bach , and D. Pfister , “Palliative Chirurgie des metastasierten Prostatakarzinoms,” Urologie 63 (2024): 241. 10.1007/s00120-024-02285-8 38418597

[34] W. N. Brennen , C. Le Magnen , S. Karkampouna , et al., “Defining the Challenges and Opportunities for Using Patient‐Derived Models in Prostate Cancer Research,” Prostate 84 (2024): 623–635. 10.1002/pros.24682 PMC11014775 38450798

[35] M. Waseem and B.‐D. Wang , “Organoids: An Emerging Precision Medicine Model for Prostate Cancer Research,” International Journal of Molecular Sciences 25 (2024): 1093. 10.3390/ijms25021093 PMC10816550 38256166

[36] X. Chen , X. Liu , Q.‐H. Li , et al., “A Patient‐Derived Organoid‐Based Study Identified an ASO Targeting SNORD14E for Endometrial Cancer Through Reducing Aberrant FOXM1 Expression and β‐Catenin Nuclear Accumulation,” Journal of Experimental & Clinical Cancer Research 42 (2023): 230. 10.1186/s13046-023-02801-2 PMC10478245 37667311

[37] J. Lange , H. Zhou , and A. McTague , “Cerebral Organoids and Antisense Oligonucleotide Therapeutics: Challenges and Opportunities,” Frontiers in Molecular Neuroscience 15 (2022): 941528. 10.3389/fnmol.2022.941528 PMC9274522 35836547

[38] J. C. Means , A. L. Martinez‐Bengochea , D. A. Louiselle , et al., “Rapid and Scalable Personalized ASO Screening in Patient‐Derived Organoids,” Nature 638 (2025): 237–243. 10.1038/s41586-024-08462-1 PMC11798851 39843740

[39] P. J. Schweiger and K. B. Jensen , “Modeling Human Disease Using Organotypic Cultures,” Current Opinion in Cell Biology 43 (2016): 22–29. 10.1016/j.ceb.2016.07.003 27474805

[40] S. T. Crooke , S. Wang , T. A. Vickers , W. Shen , and X.‐H. Liang , “Cellular Uptake and Trafficking of Antisense Oligonucleotides,” Nature Biotechnology 35 (2017): 230–237. 10.1038/nbt.3779 28244996

[41] R. L. Juliano , “The Delivery of Therapeutic Oligonucleotides,” Nucleic Acids Research 44 (2016): 6518–6548. 10.1093/nar/gkw236 PMC5001581 27084936

[42] S. Karaki , S. Benizri , R. Mejías , et al., “RETRACTED: Lipid‐Oligonucleotide Conjugates Improve Cellular Uptake and Efficiency of TCTP‐Antisense in Castration‐Resistant Prostate Cancer,” Journal of Controlled Release 258 (2017): 1–9. 10.1016/j.jconrel.2017.04.042 28472637

[43] T. C. Roberts , R. Langer , and M. J. A. Wood , “Advances in Oligonucleotide Drug Delivery,” Nature Reviews Drug Discovery 19 (2020): 673–694. 10.1038/s41573-020-0075-7 PMC7419031 32782413

[44] R. L. Juliano , “Intracellular Trafficking and Endosomal Release of Oligonucleotides: What We Know and What We Don't,” Nucleic Acid Therapeutics 28 (2018): 166. 10.1089/nat.2018.0727 PMC5994677 29708838

[45] R. A. M. Buijsen , L. M. van der Graaf , E. C. Kuijper , et al., “Calcium‐Enhanced Medium‐Based Delivery of Splice Modulating Antisense Oligonucleotides in 2D and 3D hiPSC‐Derived Neuronal Models,” Biomedicines 12 (2024): 1933. 10.3390/biomedicines12091933 PMC11428300 39335447

[46] X. Ye , S. Chen , W. Xiong , et al., “Magnetic‐Guided Delivery of Antisense Oligonucleotides for Targeted Transduction in Multiple Retinal Explant and Organoid Models,” Advanced Science 12 (2025): 2417363. 10.1002/advs.202417363 PMC12165039 40278802

[47] E. Fujii , M. Yamazaki , S. Kawai , et al., “A Simple Method for Histopathological Evaluation of Organoids,” Journal of Toxicologic Pathology 31 (2018): 81–85. 10.1293/tox.2017-0060 PMC5820108 29479145

