# Supplementary material for: Prostate Cancer Patient‐Derived Organoids (PDOs) as Functional Avatars for Antisense Oligonucleotide (ASO)‐Based Personalized Therapy
Source: Adv Healthc Mater. 2026 Oct 11;15(41):e05251. doi: 10.1002/adhm.202505251 (PMC13740942; doi:10.1002/adhm.202505251)
Supplement: Supplementary file 1 [file ADHM-15-0-s001.docx]

**Supporting Information**

**Table S1: Clinical and pathological characteristics of the patients included in the PC biobank**

| Patient ID | Pathology | Gleason score | Tissue source | PSA | Previous treatments | Next treatment lines | NGS |
| --- | --- | --- | --- | --- | --- | --- | --- |
| Patient #1 | BPH | no tumor | TURP |  |  |  |  |
| Patient #2 | BPH | no tumor | TURP |  |  |  |  |
| Patient #3 | CSPC | 9 (4+5) | TURP | 363 ng/mL | No | LHRH antagonist + abiraterone + docetaxel | TP53 mutation |
| Patient #4 | BPH |  | TURP | 39.9 ng/mL | LHRH analogue + enzalutamide | Docetaxel, ¹⁷⁷Lu-PSMA, cabazitaxel | NA |
| Patient #5 | CRPC | 9 (4+5) | TURP | 24 ng/mL | LHRH agonist | LHRH agonist | NA |
| Patient #6 | BPH |  | TURP |  |  |  |  |
| Patient #7 | BPH |  | TURP |  |  |  |  |
| Patient #8 | BPH |  | TURP |  |  |  |  |
| Patient #9 | CSPC | 9 (5+4) | TURP | 983 ng/mL | No | LHRH agonist | No mutation |
| Patient #10 | BPH |  | TURP |  |  |  |  |
| Patient #11 | CSPC | 9 (4+5) | TURP | 104 ng/mL | No | LHRH antagonist + abiraterone + docetaxel | No mutation |
| Patient #12 | BPH |  | TURP |  |  |  |  |
| Patient #13 | CSPC | 9 (5+4) | TURP | 360 ng/mL | No | LHRH antagonist + abiraterone | PIK3CA and TP53 mutations |
| Patient #14 | BPH |  | TURP |  |  |  |  |
| Patient #15 | BPH |  | TURP |  |  |  |  |
| Patient #16 | CSPC | 10 (5+5) | TURP | 45 ng/mL | No | LHRH antagonist | No mutation |
| Patient #17 | BPH |  | TURP |  |  |  |  |
| Patient #18 | BPH |  | TURP |  |  |  |  |
| Patient #19 | CSPC | 9 (5+4) | TURP | 1100 ng/mL | No | LHRH antagonist | No mutation |
| Patient #20 | CSPC | 10 (5+5) | Biopsy | 15 ng/mL | No | Declined treatment | No mutation |
| Patient #21 | CSPC | 10 (5+5) | Biopsy | 45 ng/mL | No | LHRH antagonist + apalutamide | TP53 mutation |
| Patient #22 | CSPC | 10 (5+5) | Biopsy | 60 ng/mL | No | LHRH antagonist | NA |
| Patient #23 | CSPC | 7 (4+3) | TURP | 727 ng/mL | No | LHRH antagonist | NA |
| Patient #24 | CSPC | 9 (5+4) | TURP | 240 ng/mL | No | LHRH antagonist, darolutamide, docetaxel | CHEK2 mutation |
| Patient #25 | CSPC | 10 (5+5) | TURP | 40 ng/mL | No | Declined treatment |  |
| Patient #26 | CSPC | 10 (5+5) | TURP | 86 ng/mL | No | LHRH antagonist + apalutamide | No mutation |
| Patient #27 | CSPC |  | Biopsy | 371 ng/mL | No | ADT + Abiraterone + docetaxel | NA |

**Footnote:** Benign Prostatic Hyperplasia (BPH); Castration-Sensitive PC (CSPC); Castration-Resistant PC (CRPC); Transurethral Resection of the prostate (TURP); Next-Generation Sequencing (NGS); Not available (NA). Patient #4: benign (BPH) tissue obtained by TURP from a patient with a history of treated castration-resistant PC; this patient was therefore excluded from the RT-qPCR comparison in Figure 4C.

**Table S2: Culture and maintenance of patient-derived organoids (PDOs)**

| Patient ID | Pathology | Tissue source | Passage 0 | Passage 1 | Passage 2 | Passage 3 | Passage 5 | Thaw |
| --- | --- | --- | --- | --- | --- | --- | --- | --- |
| Patient #1 | BPH | TURP | N/A | N/A | N/A | N/A | N/A | N/A |
| Patient #2 | BPH | TURP | N/A | N/A | N/A | N/A | N/A | N/A |
| Patient #3 | CSPC | TURP | + | + | N/A | N/A | N/A | Failed |
| Patient #4 | BPH | TURP | +++ | ++ | + | + | N/A | ++ |
| Patient #5 | CRPC | TURP | ++ | ++ | + | + | N/A | + |
| Patient #6 | BPH | TURP | +++ | +++ | ++ | ++ | + | ++ |
| Patient #7 | BPH | TURP | ++ | ++ | ++ | ++ | + | ++ |
| Patient #8 | BPH | TURP | +++ | +++ | +++ | ++ | ++ | ++ |
| Patient #9 | CSPC | TURP | ++ | + | + | + | N/A | + |
| Patient #10 | BPH | TURP | ++ | ++ | ++ | ++ | N/A | ++ |
| Patient #11 | CSPC | TURP | +++ | +++ | ++ | + | N/A | + |
| Patient #12 | BPH | TURP | N/A | N/A | N/A | N/A | N/A | N/A |
| Patient #13 | CSPC | TURP | Failed | N/A | N/A | N/A | N/A | - |
| Patient #14 | BPH | TURP | N/A | N/A | N/A | N/A | N/A | N/A |
| Patient #15 | BPH | TURP | +++ | +++ | ++ | ++ | N/A | ++ |
| Patient #16 | CSPC | TURP | +++ | +++ | ++ | ++ | + | ++ |
| Patient #17 | BPH | TURP | N/A | N/A | N/A | N/A | N/A | N/A |
| Patient #18 | BPH | TURP | N/A | N/A | N/A | N/A | N/A | N/A |
| Patient #19 | CSPC | TURP | +++ | +++ | ++ | N/A | N/A | ++ |
| Patient #20 | CSPC | Biopsy | + | + | N/A | N/A | N/A | N/A |
| Patient #21 | CSPC | Biopsy | + | + | N/A | N/A | N/A | N/A |
| Patient #22 | CSPC | Biopsy | + | + | N/A | N/A | N/A | N/A |
| Patient #23 | CSPC | TURP | +++ | +++ | ++ | N/A | N/A | ++ |
| Patient #24 | CSPC | TURP | +++ | +++ | ++ | N/A | N/A | ++ |
| Patient #25 | CSPC | TURP | ++ | ++ | ++ | N/A | N/A | N/A |
| Patient #26 | CSPC | TURP | +++ | ++ | ++ | N/A | N/A | N/A |
| Patient #27 | CSPC | Biopsy | Failed | N/A | N/A | N/A | N/A | N/A |

**Footnote:** Benign Prostatic Hyperplasia (BPH); Castration-Sensitive PC (CSPC); Castration-Resistant PC (CRPC); High organoid formation efficiency (OFE) with more than 100 organoids/dome (+++); medium OFE with 50–100 organoids/dome (++); low OFE with fewer than 50 organoids/dome (+)

**Table S3: Antibodies used in this study**

| **Primary antibodies** | | | | |
| --- | --- | --- | --- | --- |
| **Antigen** | **Type** | **Supplier** | **Catalog #** | **Dilution** |
| CK5 | Mouse mAb | Proteintech | 66727-1-Ig | IF 1:200 |
| CK8 | Rabbit pAb | Proteintech | 17514-1-AP | IF 1:200 |
| AR | Rabbit mAb | Cell Signaling Technology | 5153S | IF, IHC 1:200 |
| PSMA | Mouse mAb | Novus Biologicals | NBP1-45057 | IHC 1:200 |
| PSA | Rabbit pAb | Proteintech | 10679-1-AP | IHC 1:200 |
| AMACR | Rabbit pAb | Atlas Antibodies | HPA020912-100 | IHC 1:100 |
| PTEN | Mouse mAb | Proteintech | 60300-1-Ig | IHC 1:100 |
| HSP27 | Rabbit pAb | Enzo Life Sciences | ADI-SPA-803-F | WB 1:10000 |
| STEAP1 | Mouse mAb | Santa Cruz | sc-271872 | IHC 1:200 |
| EGF | Mouse mAb | Santa Cruz | sc-374255 | IHC 1:200 |
| DDX5 | Mouse mAb | Santa Cruz | sc-365164 | IHC 1:100 |
| DDX5 | Rabbit pAb | Abcam | ab21696 | WB 1:5000 |
| TCTP | Rabbit mAb | Abcam | ab133568 | IHC 1:200; WB 1:1000 |
| **Secondary antibodies** | | | | |
| **Antigen** | **Conjugate** | **Supplier** | **Catalog #** | **Dilution** |
| Goat anti-mouse IgG | Alexa Fluor 546 | Invitrogen | A11003 | 1:500 |
| Goat anti-rabbit IgG | Alexa Fluor 488 | Invitrogen | A11008 | 1:500 |
| Goat anti-mouse IgG | Biotin | Invitrogen | 31800 | 1:1000 |
| Goat anti-rabbit IgG | Biotin | Invitrogen | 65-6140 | 1:2000 |

**Table S4: Organoid medium composition**

| **Compound** | **Supplier** | **Catalog #** | **Solvent** | **Concentration** |  |
| --- | --- | --- | --- | --- | --- |
|  |  |  |  |  |  |
| B27 | Gibco | 17504-044 | ready to use | 1X |  |
| N-Acetyl-cysteine | Sigma-Aldrich | A9165 | H2O | 1.25 mM |  |
| Noggin | Peprotech | 120-10C | DMEM/F12 +++ | 100 ng/ml |  |
| R-spondin-1 | Peprotech | 120-38 | D-PBS 0.1% BSA | 500 ng/ml |  |
| A83-01 | Sigma-Aldrich | SML0788 | DMSO | 500 nM |  |
| EGF | Peprotech | AF-100-15 | PBS 0.1% BSA | 5 ng/ml |  |
| FGF-10 | Peprotech | 100-26 | PBS 0.1% BSA | 10 ng/ml |  |
| FGF-2 (FGF-basic) | Peprotech | 100-18B | PBS 0.1% BSA | 5 ng/ml |  |
| Prostaglandin E2 | Tocris Bioscience | 2296 | DMSO | 1 µM |  |
| Nicotinamide | Sigma-Aldrich | N0636 | D-PBS | 10 mM |  |
| SB202190 | Sigma-Aldrich | S7076 | DMSO | 10 µM |  |
| Dihydrotestosterone (DHT) | Sigma-Aldrich | A-8380 | Ethanol absolute | 1 nM |  |
| Y-27632 dihydrochloride | Bio-techne | 1254 | DMSO | 10 µM |  |

**Table S5: Sequences of primers and probes**

| **Genes** | S**equences** |
| --- | --- |
| **HSP27** | Fw: GAGATCACCGGCAAGCAC  Rv: ACAGGGAGGAGGAAACTTGG  Probe: AGCCATGCTCGTCCTGCCGC |
| **GAPDH** | Fw: ACCATCTTCCAGGAGCGAGA  Rv: ATGGTGGTGAAGACGCCAGT  Probe: ACTCAGCGCCAGCATCGCCC |

| **CHEK2** | Fw: CTTCTATTGCCTTCCACTTCA  Rv: AAACTCCAGCCAGTCCT |
| --- | --- |
